# Supplementary material for: Twenty-year trends in antimicrobial resistance from aquaculture and fisheries in Asia
Source: Nat Commun. 2021 Sep 10;12:5384. doi: 10.1038/s41467-021-25655-8 (PMC8433129; doi:10.1038/s41467-021-25655-8)
Supplement: Supplementary file 1 — Supplementary Information [file 41467_2021_25655_MOESM1_ESM.pdf]

Supplementary Information for

Twenty-year trends in antimicrobial resistance from aquaculture and fisheries in Asia

Daniel Schar [a,*,1], Cheng Zhao [b], Yu Wang [b], D.G. Joakim Larsson [c,d], Marius Gilbert [a,e,2], Thomas P. Van Boeckel [b,f,*,1,2]

[a]Spatial Epidemiology Laboratory, Université Libre de Bruxelles, B1050 Brussels, Belgium;

[b]Institute for Environmental Decisions, ETH Zurich, Zurich 8006, Switzerland;

[c]Center for Antibiotic Resistance Research, University of Gothenburg, Sweden;

[d]Department of Infectious Diseases, Institute for Biomedicine, University of Gothenburg, Sweden;

[e]Fonds National de la Recherche Scientifique, B1000 Brussels, Belgium;

[f]Center for Diseases Dynamics, Economics, and Policy, New Delhi, India.

[1]Co-corresponding authors

[2]MG and TPVB supervised the work equally.

[*]To whom correspondence may be addressed.

Daniel Schar, Unit 7201 Box 692

DPO AE 09974-0692

Email: dlschar@gmail.com

Thomas P. Van Boeckel

Email: thomas.vanboeckel@env.ethz.ch

This file includes:

Supplementary Notes 1 to 4
Figs. S1 to S19
Tables S1 to S4
Supplementary References

Supplementary Note 1: Systematic Review and Literature Search

A systematic review of the literature was conducted to identify point prevalence surveys (PPS) of phenotypic antimicrobial resistance inclusive of the period January 1, 2000 through September 30, 2019. Eligibility criteria included: PPS of antimicrobial resistance in bacterial pathogens of production significance or aquatic animal-associated bacterial zoonoses; PPS with samples originating from cultured or wild caught aquatic animals or their products; PPS conducted in Asia from 2000 to 2019. Target bacterial pathogens were defined according to OIE Aquatic Animal Health Code[1] and the FAO List of Important Bacterial Diseases in Aquaculture, and included: *Aerococcus*, *Aeromonas*, *Chlamydia*, *Clostridium*, *Edwardsiella*, *Enterobacterium*, *Escherichia coli*, *Flavobacterium*, *Francisella*, *Hepatobacter*, *Lactococcus*, *Mycobacterium*, *Nocardia*, *Photobacterium*, *Piscirickettsia*, *Pseudomonas*, *Renibacterium*, *Streptococcus*, *Vibrio*, and *Yersinia*.

Exclusion criteria included: reviews; meta-analyses; strain surveys describing individual strain characteristics not associated with a PPS; data from an experimental protocol not associated with a PPS; PPS with samples originating from bivalve molluscs; PPS with samples originating from ornamental fish; and PPS where no source or methodology for derivation of data was provided. We assessed data quality in our search, excluding records where resistance rates were unclear or missing; no geographic information on survey location was provided; samples originated from imported products; or samples were not clearly identified as originating from an aquatic animal or animal product.

The literature search was conducted across four databases (PubMed, Web of Science, Scopus, China National Knowledge Infrastructure) and grey literature repositories (AGRIS, CGIAR FISH, IFPRI, WorldFish). The Russian Science Citation Index and Korean Journal Database (KCI) were included in the Web of Science search. The search was conducted through September 2019 without restriction as to language, and records in English, Turkish, Japanese, Korean, Thai, and Chinese were identified and screened. The systematic review was not registered. The literature search and systematic review were guided by the Preferred Reporting Items for Systematic reviews and Meta-Analyses (PRISMA) statement and research synthesis norms[2] (Table S4).

Search strategies were tailored to individual databases to achieve optimal search sensitivity and specificity guided by the following general formula:

(Resistance) AND (Bacteria species) AND (Aquaculture/fisheries/aquatic animals) AND (Country/region)

The full search queries were:
PubMed: (Resistance OR "antibiotic resistance" OR "antimicrobial resistance") AND ("Escherichia coli" OR "E. coli" OR *vibrio* OR Photobacterium OR Aeromonas* OR Edwardsiell* OR Yersinia OR Pseudomonas* OR Flavobacter* OR Piscirickettsia OR Hepatobacter OR Francisella OR Chlamydia OR Mycobacter* OR Nocardia OR Streptococc* OR Lactococc* OR Aerococc* OR Renibacter* OR Clostridium OR Enterobacterium) AND (aquaculture OR aquatic OR *fish* OR shellfish OR marine OR freshwater OR carp OR catfish OR prawn OR salmon OR shrimp OR tilapia OR trout) AND (Asia OR "Southeast Asia" OR "South Asia" OR "East Asia" OR Mekong OR Afghanistan OR "American Samoa"

OR Bahrain OR Bangladesh OR Bhutan OR "Brunei Darussalam" OR Cambodia OR China OR "Chinese Taipei" OR "Cook Islands" OR "Democratic People's Republic of Korea" OR Fiji OR "French Polynesia" OR Guam OR "Hong Kong" OR India OR Indonesia OR Iran OR Iraq OR Jordan OR Kiribati OR Korea OR Kuwait OR "Lao People's Democratic Republic" OR Lao OR Laos OR Lebanon OR Macau OR Malaysia OR Maldives OR "Marshall Islands" OR Micronesia OR Mongolia OR Myanmar OR Nauru OR Nepal OR "New Caledonia" OR Niue OR "Norfolk Island" OR "Northern Mariana Islands" OR Oman OR Pakistan OR Philippines OR Palau OR Palestine OR "Papua New Guinea" OR "Pitcairn Islands" OR Qatar OR Samoa OR "Saudi Arabia" OR Singapore OR "Solomon Islands" OR "Sri Lanka" OR "Syrian Arab Republic" OR Taiwan OR Thailand OR Timor-Leste OR Tokelau OR Tonga OR Turkey OR Tuvalu OR "United Arab Emirates" OR Vanuatu OR "Viet Nam" OR Vietnam OR "Wallis and Futuna Islands" OR Yemen)

Web of Science [All Databases, inclusive of Russian Science Citation Index and Korean Journal Database (KCI)]: TOPIC: (Resistance OR "antibiotic resistance" OR "antimicrobial resistance") AND TOPIC: ("Escherichia coli" OR "E. coli" OR *vibrio* OR Photobacterium OR Aeromonas* OR Edwardsiell* OR Yersinia OR Pseudomonas* OR Flavobacter* OR Piscirickettsia OR Hepatobacter OR Francisella OR Chlamydia OR Mycobacter* OR Nocardia OR Streptococc* OR Lactococc* OR Aerococc* OR Renibacter* OR Clostridium OR Enterobacterium) AND TOPIC: (aquaculture OR aquatic OR *fish* OR shellfish OR marine OR freshwater OR carp OR catfish OR prawn OR salmon OR shrimp OR tilapia OR trout) AND TOPIC: (Asia OR "Southeast Asia" OR "South Asia" OR "East Asia" OR Mekong OR Afghanistan OR "American Samoa" OR Bahrain OR Bangladesh OR Bhutan OR "Brunei Darussalam" OR Cambodia OR China OR "Chinese Taipei" OR "Cook Islands" OR "Democratic People's Republic of Korea" OR Fiji OR "French Polynesia" OR Guam OR "Hong Kong" OR India OR Indonesia OR Iran OR Iraq OR Jordan OR Kiribati OR Korea OR Kuwait OR "Lao People's Democratic Republic" OR Lao OR Laos OR Lebanon OR Macau OR Malaysia OR Maldives OR "Marshall Islands" OR Micronesia OR Mongolia OR Myanmar OR Nauru OR Nepal OR "New Caledonia" OR Niue OR "Norfolk Island" OR "Northern Mariana Islands" OR Oman OR Pakistan OR Philippines OR Palau OR Palestine OR "Papua New Guinea" OR "Pitcairn Islands" OR Qatar OR Samoa OR "Saudi Arabia" OR Singapore OR "Solomon Islands" OR "Sri Lanka" OR "Syrian Arab Republic" OR Taiwan OR Thailand OR Timor-Leste OR Tokelau OR Tonga OR Turkey OR Tuvalu OR "United Arab Emirates" OR Vanuatu OR "Viet Nam" OR Vietnam OR "Wallis and Futuna Islands" OR Yemen)

Scopus: TITLE-ABS-KEY (resistance OR "antibiotic resistance" OR "antimicrobial resistance") AND TITLE-ABS-KEY ("Escherichia coli" OR "E. coli" OR *vibrio* OR photobacterium OR aeromonas* OR edwardsiell* OR yersinia OR pseudomonas* OR flavobacter* OR piscirickettsia OR hepatobacter OR francisella OR chlamydia OR mycobacter* OR nocardia OR streptococc* OR lactococc* OR aerococc* OR renibacter* OR clostridium OR enterobacterium) AND TITLE-ABS-KEY (aquaculture OR aquatic OR *fish* OR shellfish OR marine OR freshwater OR carp OR catfish OR prawn OR salmon OR shrimp OR tilapia OR trout) AND TITLE-ABS-KEY (Asia OR "Southeast Asia" OR "South Asia" OR "East Asia" OR mekong OR afghanistan OR "American Samoa" OR bahrain OR bangladesh OR bhutan OR "Brunei Darussalam" OR cambodia OR china OR "Chinese Taipei" OR "Cook Islands" OR "Democratic People's Republic of Korea" OR fiji OR "French Polynesia" OR guam OR "Hong Kong" OR india OR indonesia OR iran OR iraq OR jordan OR kiribati OR korea OR kuwait OR "Lao People's Democratic Republic" OR lao

OR laos OR lebanon OR macau OR malaysia OR maldives OR "Marshall Islands" OR micronesia OR mongolia OR myanmar OR nauru OR nepal OR "New Caledonia" OR niue OR "Norfolk Island" OR "Northern Mariana Islands" OR oman OR pakistan OR philippines OR palau OR palestine OR "Papua New Guinea" OR "Pitcairn Islands" OR qatar OR samoa OR "Saudi Arabia" OR singapore OR "Solomon Islands" OR "Sri Lanka" OR "Syrian Arab Republic" OR taiwan OR thailand OR timor-leste OR tokelau OR tonga OR turkey OR tuvalu OR "United Arab Emirates" OR vanuatu OR "Viet Nam" OR vietnam OR "Wallis and Futuna Islands" OR yemen)

CNKI: TI = (‘抗生素’ + ‘抗菌’ + ‘兽药’ + ‘兽用药’ + ‘兽用抗生素’ + ‘用药’ + ‘抗微生物’) AND TI = (escherichia + (E*coli) + coliform + vibrio + Photobacterium + Aeromonas + Edwardsiella + Edwardsiellosis + Yersinia + Pseudomonas + Flavobacter + Piscirickettsia + Hepatobacter + Francisella + Chlamydia + Mycobacter + Nocardia + Streptococcus + Lactococcus + Aerococcus + Renibacterium + Clostridium + Enterobacterium + ‘大肠菌’ + ‘埃希菌’ + ‘弧菌’ + ‘光细菌’ + ‘气单胞菌’ + ‘埃德华氏菌’ + ‘耶尔森氏菌’ + ‘假单胞菌’ + ‘黄杆菌’ + ‘立克次氏体’ + ‘肝杆菌’ + ‘弗朗西菌’ + ‘衣原体’ + ‘分枝杆菌’ + ‘诺卡氏菌’ + ‘链球菌’ + ‘乳球菌’ + ‘空气球菌’ + ‘肾菌’ + ‘梭菌’ + ‘肠杆菌’) AND TI = (‘水产’ + ‘鱼’ + ‘渔’ + ‘贝’ + ‘海’ + ‘淡水’ + ‘鲤’ + ‘虾’ + ‘鲑’ + ‘罗非’ + ‘鳟’)) OR (KY = (‘抗生素’ + ‘抗菌’ + ‘兽药’ + ‘兽用药’ + ‘兽用抗生素’ + ‘用药’ + ‘抗微生物’) AND KY = (escherichia + (E*coli) + coliform + vibrio + Photobacterium + Aeromonas + Edwardsiella + Edwardsiellosis + Yersinia + Pseudomonas + Flavobacter + Piscirickettsia + Hepatobacter + Francisella + Chlamydia + Mycobacter + Nocardia + Streptococcus + Lactococcus + Aerococcus + Renibacterium + Clostridium + Enterobacterium + ‘大肠菌’ + ‘埃希菌’ + ‘弧菌’ + ‘光细菌’ + ‘气单胞菌’ + ‘埃德华氏菌’ + ‘耶尔森氏菌’ + ‘假单胞菌’ + ‘黄杆菌’ + ‘立克次氏体’ + ‘肝杆菌’ + ‘弗朗西菌’ + ‘衣原体’ + ‘分枝杆菌’ + ‘诺卡氏菌’ + ‘链球菌’ + ‘乳球菌’ + ‘空气球菌’ + ‘肾菌’ + ‘梭菌’ + ‘肠杆菌’) AND KY = (‘水产’ + ‘鱼’ + ‘渔’ + ‘贝’ + ‘海’ + ‘淡水’ + ‘鲤’ + ‘虾’ + ‘鲑’ + ‘罗非’ + ‘鳟’)) OR (AB = (‘抗生素’ + ‘抗菌’ + ‘兽药’ + ‘兽用药’ + ‘兽用抗生素’ + ‘用药’ + ‘抗微生物’) AND AB = (escherichia + (E*coli) + coliform + vibrio + Photobacterium + Aeromonas + Edwardsiella + Edwardsiellosis + Yersinia + Pseudomonas + Flavobacter + Piscirickettsia + Hepatobacter + Francisella + Chlamydia + Mycobacter + Nocardia + Streptococcus + Lactococcus + Aerococcus + Renibacterium + Clostridium + Enterobacterium + ‘大肠菌’ + ‘埃希菌’ + ‘弧菌’ + ‘光细菌’ + ‘气单胞菌’ + ‘埃德华氏菌’ + ‘耶尔森氏菌’ + ‘假单胞菌’ + ‘黄杆菌’ + ‘立克次氏体’ + ‘肝杆菌’ + ‘弗朗西菌’ + ‘衣原体’ + ‘分枝杆菌’ + ‘诺卡氏菌’ + ‘链球菌’ + ‘乳球菌’ + ‘空气球菌’ + ‘肾菌’ + ‘梭菌’ + ‘肠杆菌’) AND AB = (‘水产’ + ‘鱼’ + ‘渔’ + ‘贝’ + ‘海’ + ‘淡水’ + ‘鲤’ + ‘虾’ + ‘鲑’ + ‘罗非’ + ‘鳟’))

Grey literature searches:

AGRIS: (Resistance OR "antibiotic resistance" OR "antimicrobial resistance") AND (bacteria species) AND (aquaculture OR aquatic OR fish OR shellfish OR marine OR freshwater OR fish spp.) AND (Asia OR "Southeast Asia" OR "South Asia" OR "East Asia" OR Mekong)

CGIAR FISH: Resistance OR "antibiotic resistance" OR "antimicrobial resistance"

IFPRI: Resistance OR "antibiotic resistance" OR "antimicrobial resistance"

WorldFish: (Resistance OR "antibiotic resistance" OR "antimicrobial resistance”) AND (Asia OR "Southeast Asia" OR "South Asia" OR "East Asia" OR Mekong)

The literature search identified 5,804 unique records screened by titles and abstracts. Of these records, 1,131 full text articles were assessed for eligibility, and 343 records yielding 749 point prevalence surveys were included in the data analysis (Fig. S1).

Supplementary Note 2: Data Extraction

Data extraction was performed according to the following protocols to generate the database used for analysis. Data was extracted using Google Sheets and Microsoft Excel (version 16.51). The protocols are adapted from *Resistancebank*[3], adjusted for the contexts and characteristics of PPS conducted from aquatic animals and settings. The data extracted from each record included the Direct Object Identifier (DOI), author, year of publication, country, latitude and longitude of sample collection, derivation of lat/long coordinates, location type of sample origin, sampling dates, animal species sampled and whether cultured or wild caught, sample type collected, health status of animal, history of antimicrobial use, pathogen and strain, number of samples collected, number of isolates subjected to antimicrobial susceptibility testing (AST), AST method, breakpoints and guidelines used, drug class, compound, resistance rate, and author contact details.

The complete database legend is available on the Zenodo public repository (doi: 10.5281/zenodo.4609884)[4].

The following broad antibiotic classes were included in the database: PEN (Penicillins), CEP (Cephalosporins), MON (Monobactams), CAR (Carbapenems), AMI (Aminoglycosides), QUI (Quinolones), AMP (Amphenicols), TET (Tetracyclines), SUL (Sulfonamides), MAC (Macrolides), Glycopeptides (GLY), POL (Polymyxins), and OTH (Others).

Antimicrobial compounds used for susceptibility testing were designated by a 3-letter code and the Anatomical Therapeutic Chemical (ATC) Classification code designation (https://www.whocc.no/atc_ddd_index/ or https://www.whocc.no/atcvet/atcvet_index/). ATC-Code starting with J0 stand for antibiotics for human systemic use while QJ01for veterinary use. For antibiotics without attributed ATC codes, a pseudo-code was constructed by using the ATC code of the molecular classification (5 or 6 characters for human and veterinary antibiotics, respectively) and adding the first character of the compound’s name separated by a - (e.g. Sarafloxacin – J01MA-S). Some ATC codes are

provided for mixture of compounds (e.g. J01RA01 for penicillins in combination with other antibacterials). Active ingredients' names were reported when commercial drugs were used.

The antibiotics found across all studies are the following (3 letter code, ATC-code): Amoxicillin-Clavulanic Acid (AMC, J01CR02); Ticarcillin-Clavulanic acid (TIM, J01CR03); Piperacillin-Tazobactam (PIT, J01CR05); Ampicillin-Sulbactam (SAM, J01CR01); Ampicillin (AMP, J01CA01); Amoxicillin (AMX, J01CA04); Amoxicillin-Sulbactam (AMS, J01CR02); Azlocillin (AZL, J01CA09); Ticarcillin (TIC, J01CA13); Cloxacillin (CLO, J01CF02); Oxacillin (OXA, J01CF04); Piperacillin (PIP, J01CA12); Flucloxacillin (FLU, J01CF05); Carbenicillin (CAR, J01CA03); Methicillin (MET, J01CF03); Penicillin (PEN, J01CE01); Mezocillin (MEZ, J01CA10); Ceftriaxone (CRO, J01DD04); Ceftazidime (CAZ, J01DD02); Cefalexin (CLX, J01DB01); Cefotaxime (CTX, J01DD01); Cefepime (FEP, J01DE01); Cefoxitin (FOX, J01DC01); Cefalotin (CFL, J01DB03); Ceftiofur (CFU, QJ01DD90); Cefuroxime (CXM, J01DC02); Cefpodoxime (CPD, J01DD13); Cefazolin (CFZ, J01DB04); Cefixime (CFM, J01DD08); Cefamandole (CMD, J01DC03); Cefoperazone (CFP, J01DD12); Moxalactam (MOX, J01DD06); Cefradine (CFR, J01DB09); Sulbactam-CFP (SFP, J01DD62); Ceftizoxime (CZM, J01DD07); Cephaloridine (CLD, J01DB02); CAZ-Clavulanic Acid (CAC, J01DD52); Cefotiam (CFT, J01DC07); Cefpimizole (CPM, J01DC-C); Cefminox (CMX, J01DC12); Cefaclor (CFC, J01DC04); Cefadroxil (CFR, J01DB05); Aztreonam (ATM, J01DF01); Imipenem (IPM, J01DH51); Ertapenem (ERT, J01DH03); Meropenem (MEM, J01DH02); Kanamycin (KAN, J01GB04); Gentamicin (GEN, J01GB03); Neomycin (NEO, J01GB05); Streptomycin (STR, J01GA01); Amikacin (AMK, J01GB06); Tobramycin (TOB, J01GB01); Apramycin (APR, QA07AA92); Netilmicin (NET, J01GB07); Spectinomycin (SPT, J01XX04); Fleroxacin (FLR, J01MA08); Enoxacin (ENO, J01MA04); Ciprofloxacin (CIP, J01MA02); Nalidixic acid (NAL, J01MB02); Pipemidic acid (PIM, J01MB04); Enrofloxacin (ENR, QJ01MA90); Norfloxacin (NOR, J01MA06); Ofloxacin (OFX, J01MA01); Oxolinic Acid (OXO, J01MB05); Flumequine (FLQ, J01MB07); Moxifloxacin (MXF, J01MA14); Levofloxacin (LVX, J01MA12); Pefloxacin (PEF, J01MA03); Marbofloxacin (MRB, QJ01MA93); Gatifloxacin (GAT, S01AE0E); Lomefloxacin (LOM, J01MA07); Danofloxacin (DAN, QJ01MA92); Sarafloxacin (SAR, J01MA-S); Chloramphenicol (CHL, J01BA01); Florfenicol (FFC, QJ01BA90); Thiamphenicol (TFC, J01BA02); Tetracycline (TET, J01AA07); Oxytetracycline (OXT, J01AA06); Doxycycline (DOX, J01AA02); Minocycline (MIN, J01AA08); Chlortetracycline (CTE, J01AA03); Sulfamethoxazole-Trimethoprim (SXT, J01EE01); Sulfamethoxazole (SMZ, J01EC01); Sulfafurazole or Sulfisoxazole (SOX, J01EB05); Sulfadiazine (SUD, J01EE-S); Sulfonamides (SSS, J01E); Trimethoprim-Sulfadiazine (TDZ, QJ01EW10); Trimethoprim (TMP, J01EA01); Sulfamonomethoxine (SMN, QJ01EQ18); Kitasamycin (KIT, QJ01FA93); Erythromycin (ERY, J01FA01); Oleandomycin (OLD, J01FA05); Lincomycin (LIN, J01FF02); Clindamycin (CLI, J01FF01); Clarithromycin (CLR, J01FA09); Tylosin (TYL, QJ01FA90); Azithromycin (AZM, J01FA10); Spiramycin (SPI, J01FA02); Tilmicosin (TIL, QJ01FA91); Roxithromycin (ROX, J01FA06); Midecamycin (MID, J01FA03); Vancomycin (VAN, J01XA01); Teicoplanin (TEC, J01XA02); Polymyxin B (PMB, J01XB02); Colistin (CST, J01XB01); Linezolid (LIZ, J01XX08); Nitrofurantoin (NIT, J01XE01); Nitrofurazone (NFZ, D08AF01); Bleomycin (BLM, L01DC01); Rifampicin (RIF, J04AB02); Bacitracin (BAC, J01XX10); Fosfomycin (FOF, J01XX01); Fusidic acid (FUS, J01XC01); Metronidazole (MTD, J01XD01); Pristinamycin (PRI, J01FG01); Furazolidone (FRZ, QJ01XE90); Novobiocin (NOV, QJ01XX95); Bicyclomycin (BCM, J01-B); Virginiamycin (VRG, D06AX10).

The median of the interval between mid-point of sampling dates and year of publication—set to mid-point of year (e.g. July 2nd)—was calculated for all records where such details were given. In records where sampling dates were not provided, the estimated sampling dates were then calculated by subtracting the median interval in days from the publication date.

For records requiring clarification or with missing data, corresponding authors were contacted. A total of 44 emails were sent requesting clarification, and 15 responses were received. Records were excluded when no response was received and missing or unclear data precluded further analysis.

Asia sub-regions and their corresponding countries and territories were defined according to United Nations classifications (https://unstats.un.org/unsd/methodology/m49/) (Fig. S3), with modification to merge Western Asia and Southern Asia into a single region for analysis.

There were 104 unique species or groups of species represented in our dataset. To facilitate analysis, species were aggregated into six groups reflective of aquatic animal and type of aquatic environment: marine fish, freshwater fish, brackish water fish, shrimp, and a mixed group where aquatic animal species sampled spanned categories and for which resistance rates were not disaggregated. The remaining species were pooled into a sixth group that included other crustaceans (crab), cephalopods (squid), gastropod molluscs (abalone), amphibians (frogs and salamanders), echinoderms (sea cucumbers and sea urchins), and reptiles (turtles).

Supplementary Note 3: Data Analysis

The percentage of antimicrobial compounds in each survey with resistance exceeding 50% was calculated (P50). With the exception of select *Aeromonas* and *Flavobacterium* spp. pathogens of primarily cold water salmonid species[5], the majority of aquatic animal pathogens have neither standard interpretive criteria for susceptibility testing nor clinical breakpoints to guide therapeutic interventions in aquatic animals. In the absence of standard interpretive criteria, surveys frequently used human clinical breakpoints when available, either at the bacterial species, genera or family level. Therefore, the P50 is best used as an index of multi-drug resistance rather than indicative of expected therapeutic outcomes in aquatic animals.

Analysis of Antimicrobial Resistance Trends Across All Bacteria Isolated from Aquatic Food Animals

The P50 metric was used in the analysis of temporal trends and for geospatial modeling. We compared P50 with two additional metrics: P30 (calculated as the percentage of antimicrobial compounds in each survey with resistance exceeding 30%) and mean resistance (calculated as the total number of resistant isolates divided by the number of isolates * the number of antibiotics tested in each survey). Across all surveys, there is a positive correlation between P50 and mean resistance (Pearson's correlation coefficient = 0.9596). In cultured animals, P50 (RMSE =0.223 ; coefficient = 0.004; p = 0.633) and Mean (RMSE =0.207 ; coefficient = 0.002 ; p = 0.758) were comparable with a positive coefficient slope, whereas for P30 (RMSE =0.235 ; coefficient = -0.012; p = 0.158) the slope is negative. In wild caught animals, the Mean model fit improved (RMSE = 0.176; coefficient = -0.065; p = 0.002) when compared with P50 (RMSE = 0.235; coefficient = -0.085; p = 0.003) and P30 metrics (RMSE = 0.262; coefficient = -0.106; p

= 0.001), however the directionality of the trends is consistent and remain statistically significant across all metrics (Fig. S19). We use P50 as an index of multi-drug resistance to document the temporal and geographic trends in resistance in bacteria of aquatic animal origin intended for human consumption.

Temporal trends were analyzed by fitting regression lines using generalized linear models with quasibinomial error distribution weighted by the log of the number of isolates in each survey subjected to susceptibility testing in order to reflect uncertainty in surveys with a limited number of isolates. Root mean square error (RMSE) was used to evaluate goodness of fit for the temporal trends regression models. RMSE indicated model fits were moderate ($RMSE_{cultured}$ = 0.223; and $RMSE_{wild\ caught}$ = 0.235), consistent with both the scattered nature and scarcity of the data. The 95% confidence intervals were generated as the fitted values +/- 1.96 * standard error of the fitted value.

One-way analysis of variance (ANOVA) tests were used to analyze the significance of the difference in mean P50 across all surveys when comparing samples from (*i*) cultured and wild caught aquatic animals; (*ii*) animals with or without history of antimicrobial use prior to sampling; and (*iii*) diseased and healthy aquatic animals. One-way ANOVA tests were conducted on arcsine transformed P50 values to normalize the distributions of these proportions. The distributions of the residuals were checked visually using histograms and q-q plots and with the Shapiro-Wilk normality test. Homogeneity of variance between groups was confirmed using a Bartlett test and by examining residual plots.

Analysis of Antimicrobial Resistance in Foodborne Pathogens Isolated from Aquatic Animals

The pooled prevalence of resistance was calculated from individual pathogen-drug resistance rates to report resistance in foodborne pathogens specifically (*Vibrio* spp., *E.coli*, *Streptococcus* spp. and *Aeromonas* spp.). We analyzed antimicrobial resistance in surveys at the bacterial genera level. This level of taxonomy was completely available (no missing entries) in our database, whereas the more granular bacterial species and strain level data were either not consistently provided or could not be disaggregated (143 surveys; 19%). In 2019, the U.S. FDA National Antimicrobial Resistance Monitoring System (NARMS) initiated a pilot surveillance program for pathogens from seafood[6]. This NARMS pilot study design targets *Vibrio*, *Aeromonas* and *Enterococcus* spp. reported at the genera level. Analysis of resistance in foodborne bacteria of aquatic animal origin was guided by antimicrobial compounds of relevance for therapeutic use in human clinical settings. Resistance rates were calculated for *Vibrio* spp., *E.coli*, *Streptococcus* spp. and *Aeromonas* spp. from samples originating from marine fish, freshwater fish, and shrimp groups using The Clinical & Laboratory Standards Institute (CLSI M45 and M100) and WHO Advisory Group on Integrated Surveillance of Antimicrobial Resistance (AGISAR) pathogen-drug susceptibility testing guidelines (Table S1). The 95% confidence interval was calculated for the population proportion.

Supplementary Note 4: Geospatial modeling

P50 values from point prevalence surveys were interpolated to map AMR in freshwater and marine environments at a resolution of 0.0833 decimal degrees, or approximately 10 km at the equator.

Freshwater Protocol

Using a two-step procedure, we first trained multiple child models, and subsequently stacked model predictions for universal kriging. This stacked generalization ensemble modeling approach has been used to model population level health metrics[7] as well as the distribution of AMR in terrestrial animals[3]. Such approaches have been shown to improve overall predictive accuracy when compared with individual models[8].

The freshwater data set was replicated five times to expand values for modeling. Each survey was randomly redistributed within a discrete uncertainty range in kilometers defined as the mean of the uncertainty boundaries around the X and Y coordinates for the smallest available administrative unit or place name provided in the survey. In practice, the uncertainty range for a survey where precise sampling coordinates were provided was zero, and such surveys (n=43) were therefore not redistributed. P50 values from the expanded data set were first transformed into presence or absence of resistance using random binarization, where P50 values were compared with a random number between zero and one and classified as presence if they exceed this number or absence if they fell below this number. Next, pseudo-absence points were generated and distributed to provide additional covariate values that were not associated with presences (P50 = 0). Pseudo-absence points were sampled within a radius of 20 to 500 km from presence points using stratified random sampling proportional to the human population density to account for potential P50 observation bias in more densely populated areas. Child models contained equal numbers of true presence versus absences (true absence + pseudo absences) as balanced data sets have been shown to enhance predictive accuracy of spatial models[9].

In the first step, we trained three classes of child models: boosted regression trees (BRT)[10]; least absolute shrinkage and selection operator applied to logistic regression (LASSO-GLM)[11]; and overlapped grouped LASSO penalties for General Additive Models selection (LASSO-GAM)[12]. Models were trained to quantify the association between P50 and a set of environmental and anthropogenic covariates relevant to the freshwater environment (Fig. S13 and Table S2). Covariates were log10-transformed and resampled from their original resolution to 0.0833 decimal degrees.

Prior to model fitting, spatial sorting bias (SSB) was calculated to determine whether mean distance between sets of points and their nearest reference points for the training and validation data sets for each spatial fold differed[13]. The SSB approached one (SSB = .95), indicating negligible bias. The BRT model was fit using a tree complexity of two, a learning rate of 0.0001, and a step size of 60, controlling interactions between variables, the weights of each individual tree in the final model, and the number of trees added at each cycle, respectively. All child models were fit using three-fold spatial cross validation to prevent local overfitting and bootstrapped 10 times to account for variability introduced in the redistribution of surveys according to their geographic uncertainty range; in the random binarization of P50 values; and in the stratified random sampling of pseudo-absence points. For all child models, the modified UN Asia sub-regions served as the three cross validation regions: Eastern Asia; Western and Southern Asia; and South-eastern Asia. Model predictive accuracy was evaluated by taking the mean value of the area under the receiver operator characteristic curve (AUC) for all bootstrap runs. The BRT AUC = 0.60, LASSO-GLM AUC = 0.57, and LASSO-GAM AUC = 0.56. The distribution of relative influence[10] for each covariate across all bootstraps was used to evaluate the contribution of covariates in the BRT models (Fig. S16).

In the second step, predictions from child models were stacked and used as covariates for universal kriging of P50 values between survey locations. Duplicate coordinates from surveys conducted at the same location were randomly redistributed within 1 km of the survey coordinates, weighted by the log10 of the number of isolates in the survey to reflect broader geographic range from larger surveys. We fit a Matern variogram with a cutoff of 500 km, which is where the semi-variogram attained the range. The kriging procedure was weighted by the number of isolates at each location.

We quantified spatial uncertainty in our P50 map, producing a 95% confidence interval on the predicted values (Fig. S18). We first calculated the standard deviation in predictions in each pixel in each child model. Next, we calculated a standardized kriging variance such that variance was zero at the location of observations (Fig. S17). These were used to produce the 95% confidence interval map on the predictions as follows:

$$95\%\ CI = 1.96 \times \left(sd(P_{BRT}, P_{LASSO-GLM}, P_{LASSO-GAM}) + \sqrt{Var_K}\right)$$

Where P_{BRT}, $P_{LASSO-GLM}$, $P_{LASSO-GAM}$ are the P50 predicted values from each child model and Var_K is the standardized kriging variance after stacking.

Predictions were masked to freshwater using a hybrid of the Global Lakes and Wetlands Database[14], the HydroSHEDS lakes database[15] of freshwater lakes with surface area greater than 10 hectares, and the HydroSHEDS rivers database[16] of rivers with long-term average discharge greater than 10 m^3/s.

Marine Protocol

A root mean square error (RMSE)-weighted ensemble model was used to map AMR in marine environments. In the marine model, P50 values from inland freshwater surveys were excluded. Surveys from wild caught marine animals sampled at land based post-harvest sites were randomly assigned coordinates to open ocean within a radius of .54 to 81 nautical miles (1 to 150 km) from their nearest coastal location. This range captures a distance intended to include the largest distribution of both artisanal and industrial fishing fleets in Asia, and falls within the 200 nautical mile distance from coastline exclusive economic zones for which countries retain rights to explore their marine resources. The marine data set consisted of these surveys combined with surveys originating from marine, coastal marine, brackish water, and coastal brackish water sampling locations, which were randomly distributed within 1 km radius from their original sampling location to avoid duplicate coordinates. Coastal marine surveys with land based coordinates were redistributed to their nearest coastline.

The combined marine data set was then used to interpolate P50 using only the sampling coordinates as covariates. Inverse distance weighted, natural neighbor, and ordinary kriging models were produced. In the natural neighbor model, the maximum number of neighbor locations was set to eight and the inverse distance power set to one. In the ordinary kriging model, we fit a Matern variogram model with a maximum range of 2,000 km. The kriging procedure was weighted by the number of isolates subjected to antimicrobial susceptibility testing in each survey to capture uncertainty associated with surveys with a limited number of isolates.

We then stacked the models and weighted their P50 predictions according to their root mean square error (RMSE) to capture the fit and variance of each model in the final ensemble model. The weights were taken as the inverse of the RMSE of each constituent model divided by the sum of RMSE for all models, and expressed as their relative proportion in the final RMSE-weighted marine AMR ensemble model (Table S3). A transparency function was added proportional to the spatial kernel density of surveys to reflect geographic distribution of surveys contributing to the final marine P50 map. The spatial kernel density was calculated at a distance bandwidth of 8.333 decimal degrees (approximately 1000 km at the equator), which is where the variogram of the surveys leveled or reached range.

Optimizing Locations for Future Surveillance

We identified the locations for 50 hypothetical surveys that could be conducted across Asia in the next year, aimed at maximizing the information gained from future AMR surveillance in freshwater aquaculture. Future survey locations were identified using a map of "need for surveillance" (NS), defined as:

$$NS = Var_K \cdot W_h \cdot W_a$$

Where Var_K represents kriging variance; and W_h and W_a are human population density and inland aquaculture production, respectively. These terms weight the necessity for surveillance by those areas where AMR is likely to have the greatest impact on human health and the aquaculture industry. All three terms were standardized to range [0,1], thus given equal weights in determining the need for surveillance.

We applied the approach proposed by Zhao et al. (submitted) that maximizes information gain in determining each successive survey location. First, we identified the pixel location with the highest value on the map of NS—denoted as X_p, Y_p—as the first survey location. Then the value of NS at each pixel location X_i, Y_i was recalculated as

$$NS_{(+1\ \text{survey})\ X_i,Y_i} = NS_{X_i,Y_i} \times (1 - \frac{\text{overlap area}}{\text{neighborhood area}})$$

Where the "neighborhood area" consists of all pixels within the distance of decreased *NS* to a new survey, represented as a circular area with radius *dist*. "Overlap area" is the overlapped neighborhood areas between location X_p, Y_p and X_i, Y_i. Each additional survey location was then placed successively at the pixel location with highest value on the recalculated map of NS until all surveys were assigned.

The radius *dist* of the neighborhood area was optimized using a sensitivity analysis, through approximate Bayesian computation (sequential Monte Carlo)[17], such that the sum of all pixel values on the map of NS was minimized after all survey locations were assigned and added in the kriging model. The priors of *dist* were a uniform distribution between 50 km and 500 km.

Data analysis was conducted in R version 3.6.3.

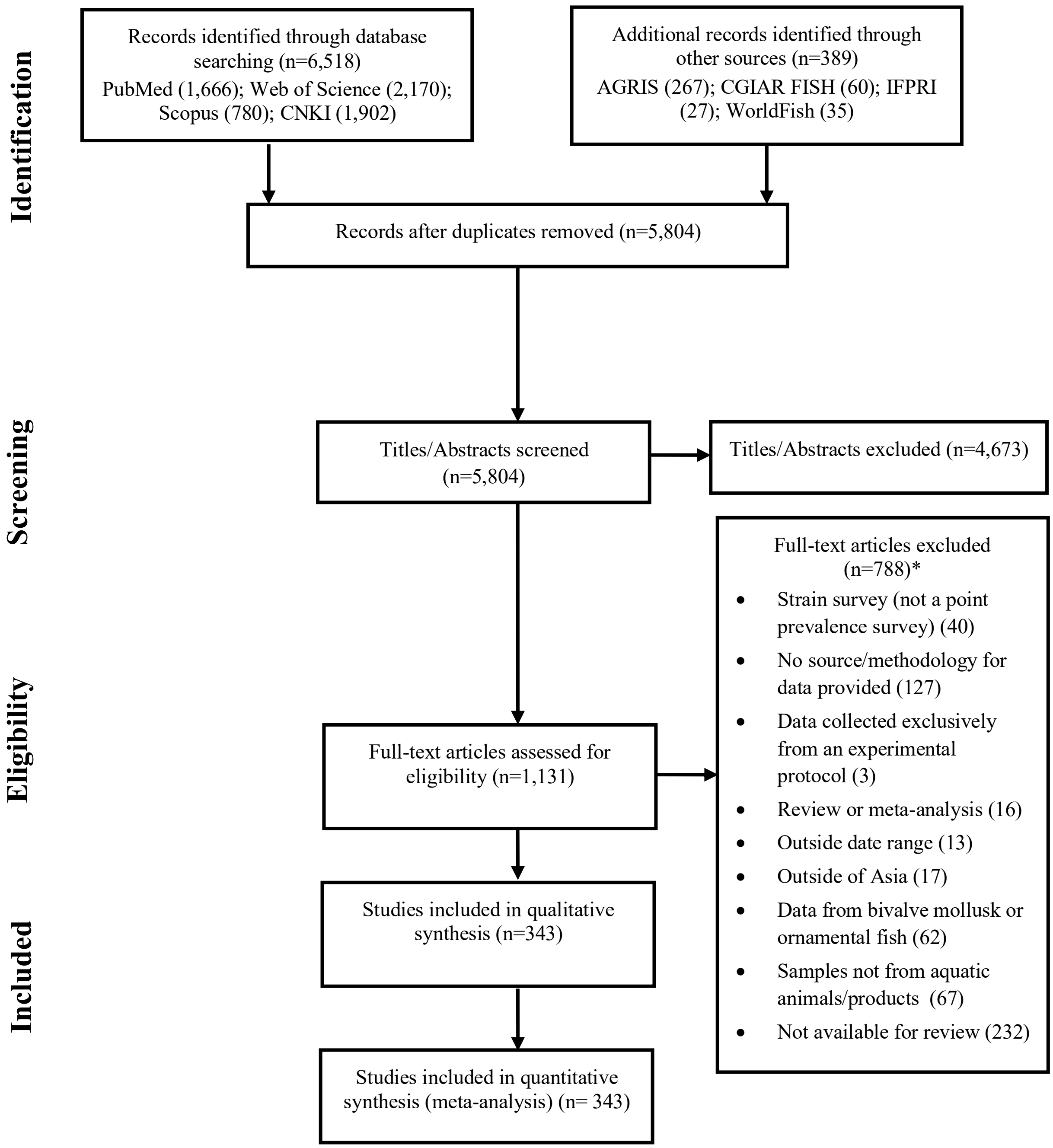

Fig. S1. Systematic review[2] and meta-analysis of antimicrobial resistance point prevalence surveys in aquatic animals. *Exclusion criteria for CNKI search records are not reflected in individual criteria totals.

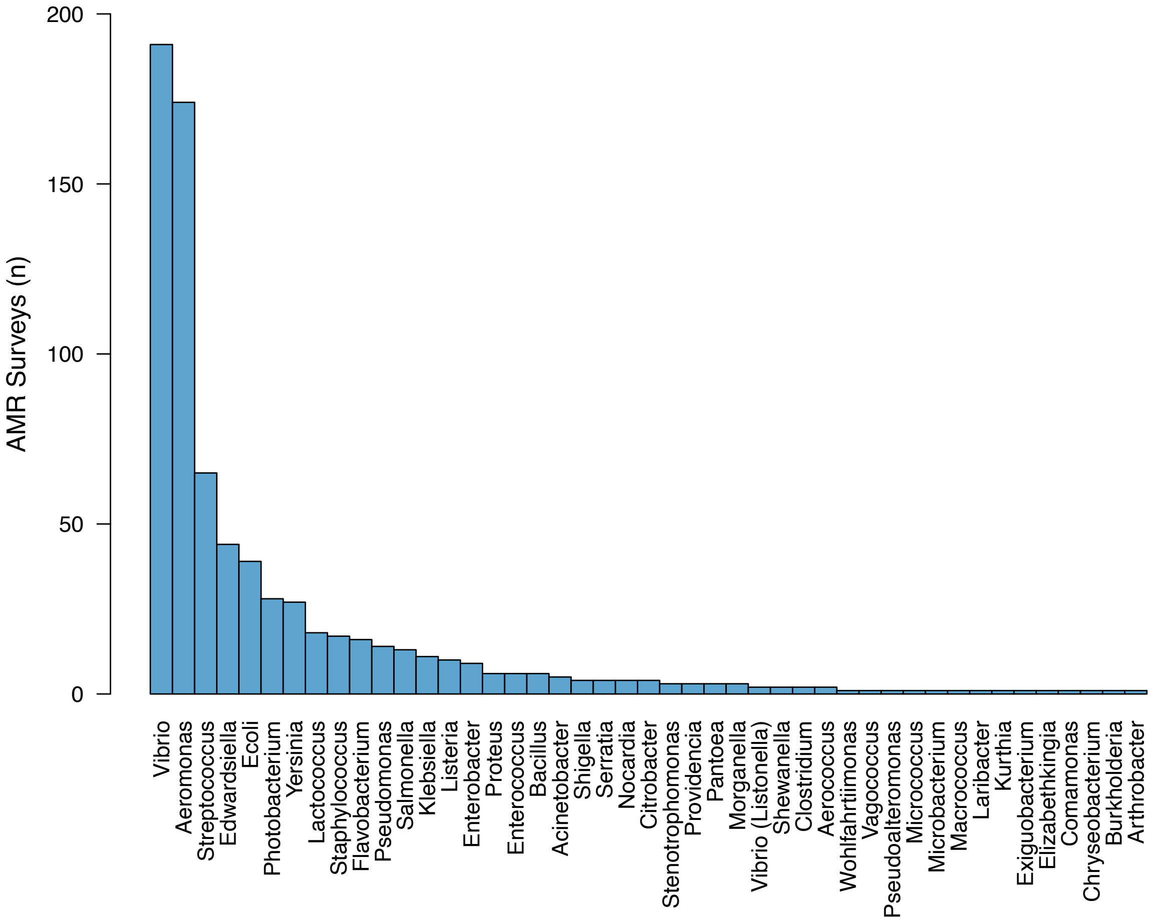

Fig. S2. Pathogens represented in resistance surveys in aquatic animals.

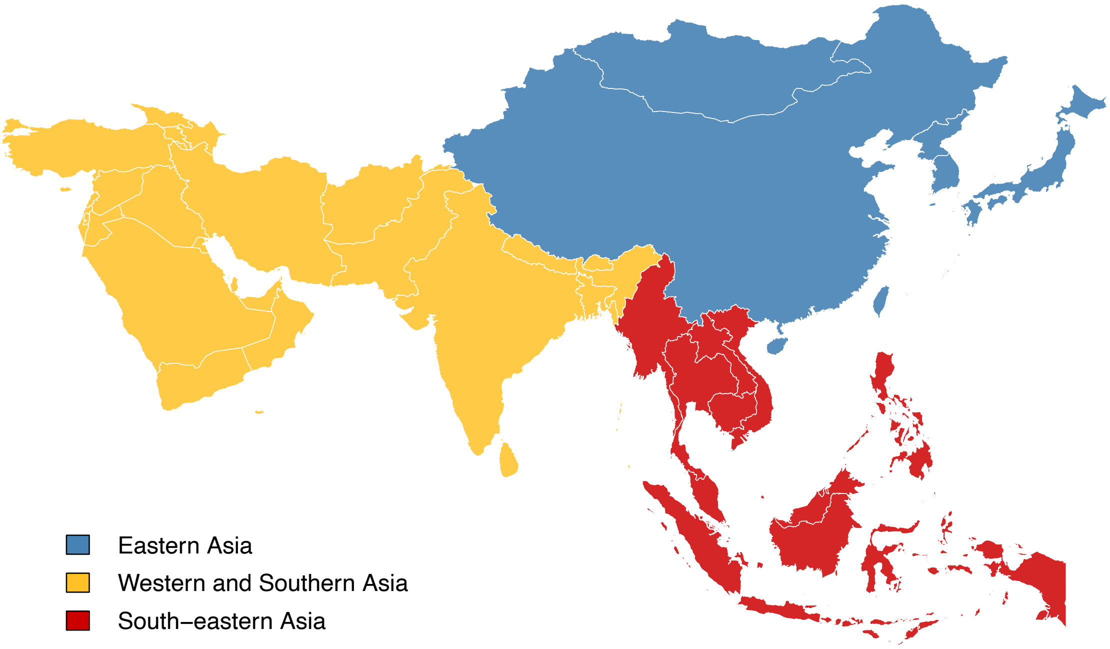

Fig. S3. Asia sub-regions from which the point prevalence surveys originate. Sub-regions are defined according to the United Nations "Standard Country or Area Codes for Statistical Use", with Western Asia and Southern Asia merged.

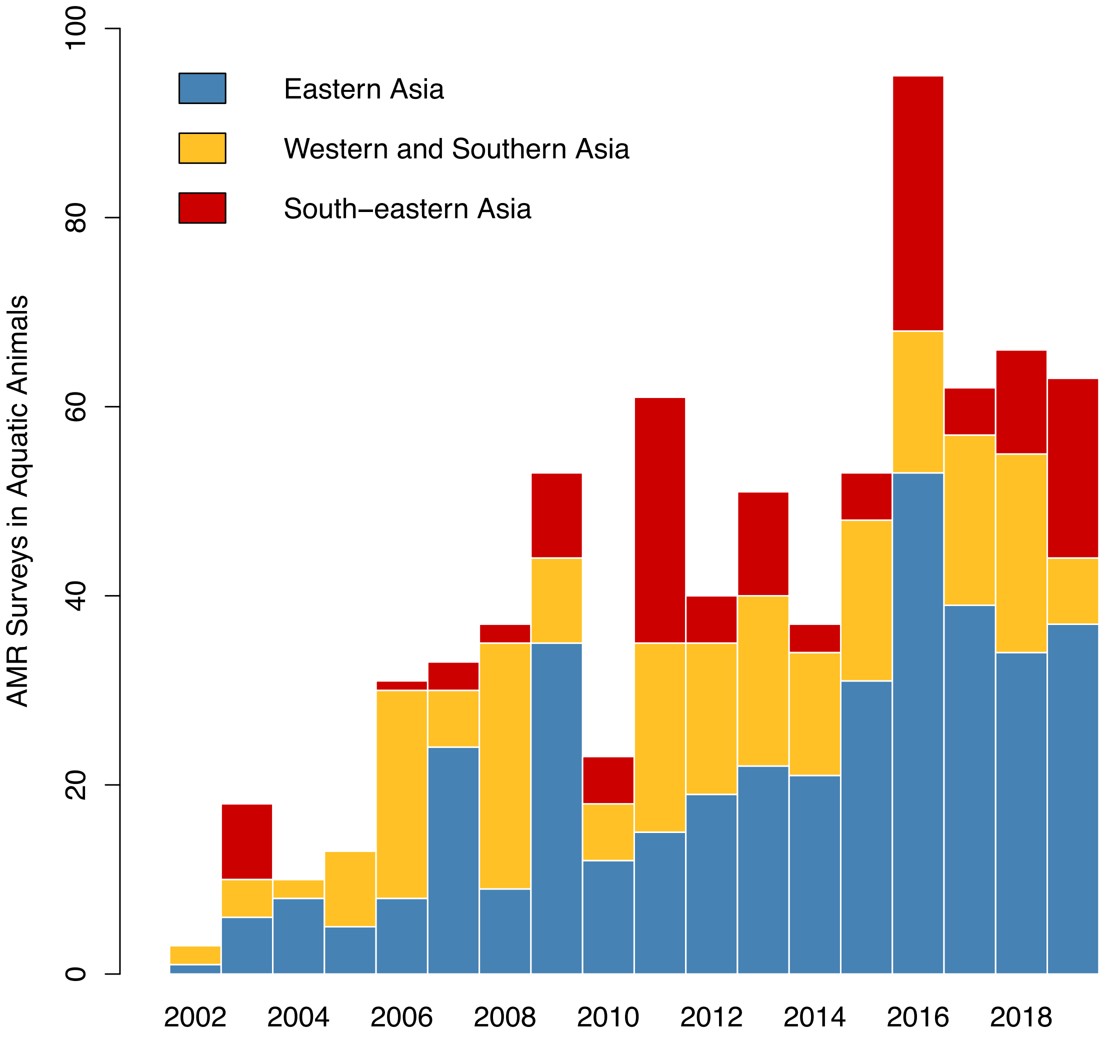

Fig. S4. Resistance surveys conducted in aquatic animals. Countries constituting each sub-region (Eastern Asia: CHN, HKG, JPN, KOR, TWN; Western and Southern Asia: BGD, IND, IRN, KWT, LBN, PAK, SAU, TUR; South-eastern Asia: KHM, MYS, PHL, SGP, THA, VNM) are represented by their International Organization for Standardization country codes. Year of publication is shown.

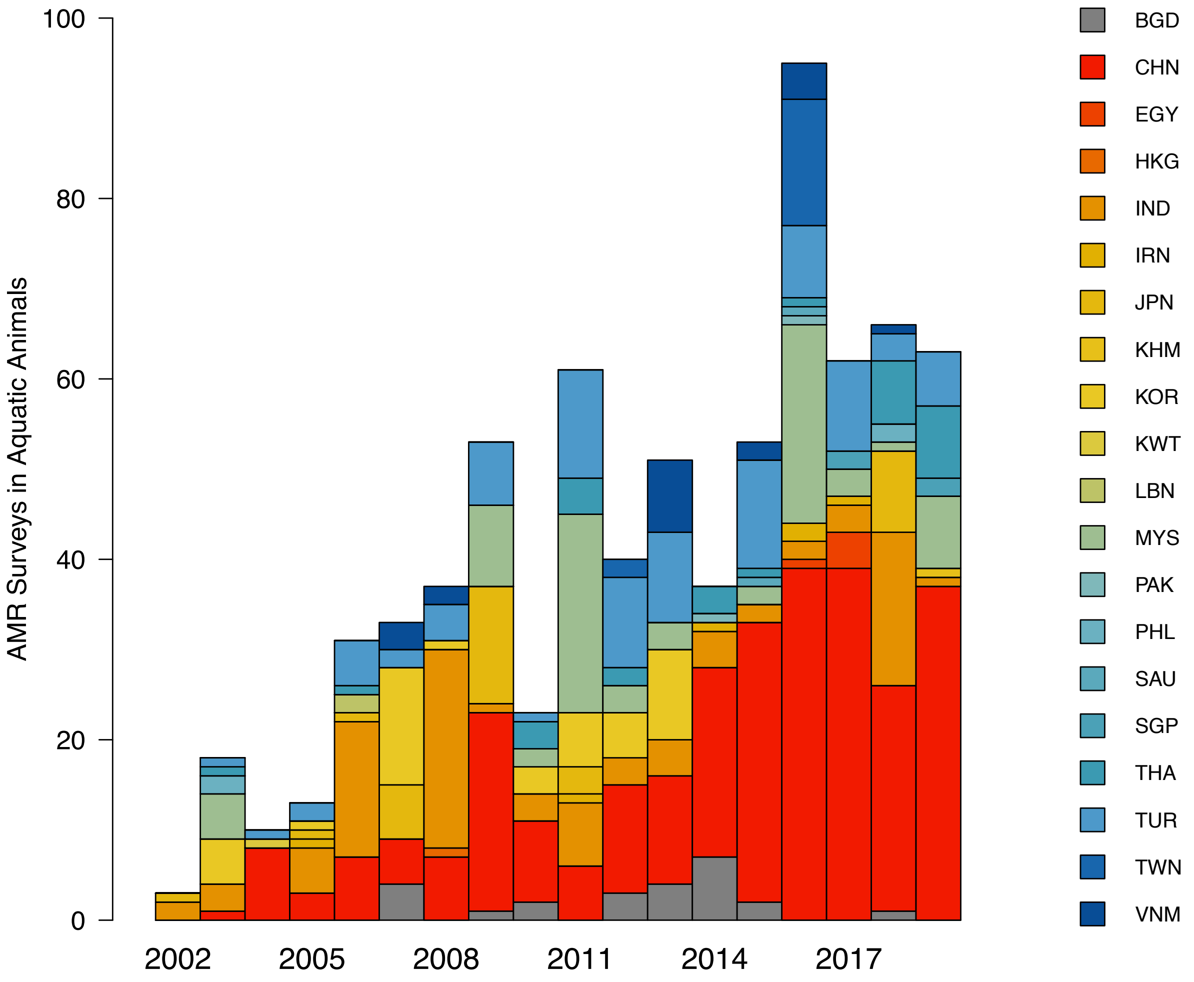

Fig. S5. Countries conducting resistance surveys in aquatic animals. Countries are represented by their International Organization for Standardization country codes. Year of publication is shown.

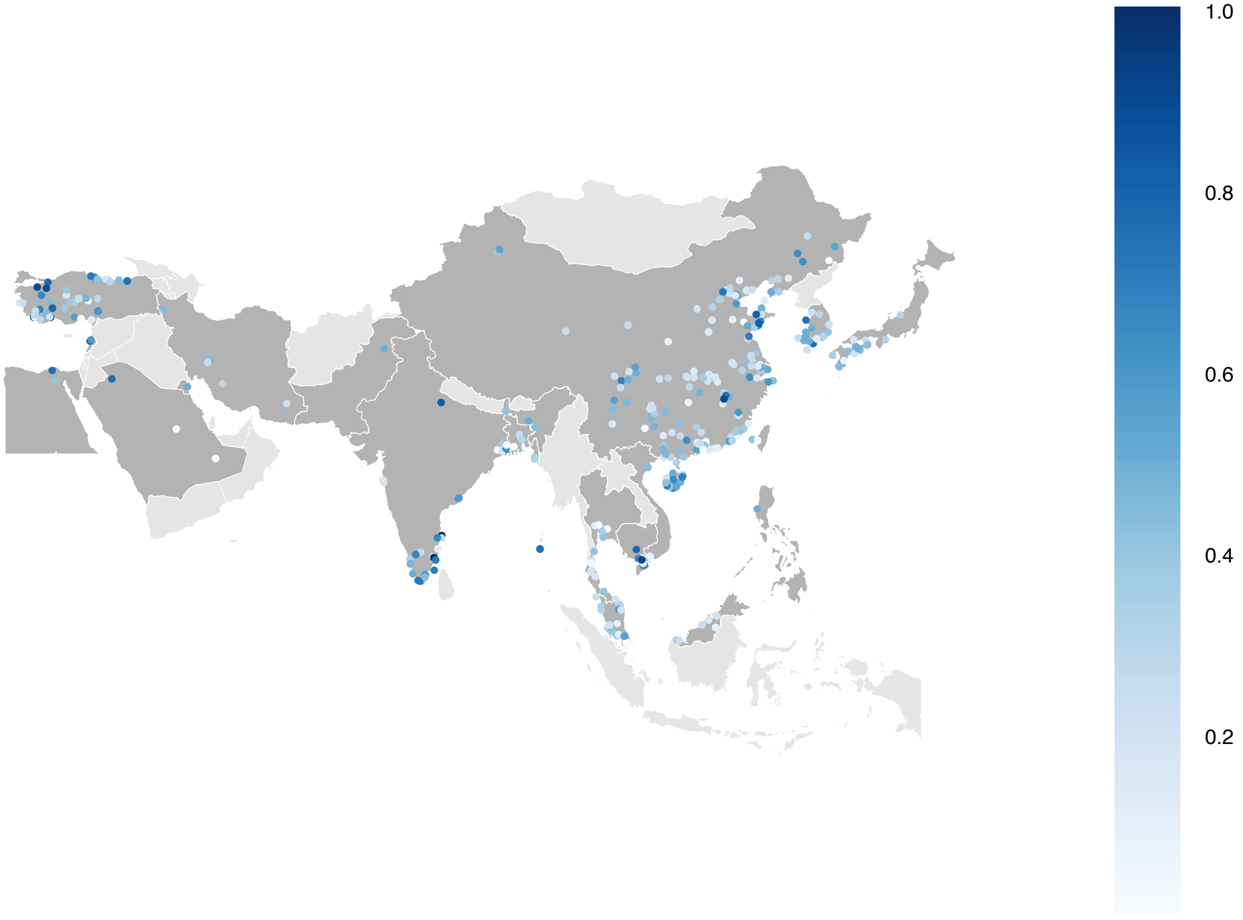

Fig. S6. Proportion of drugs with resistance higher than 50% (P50) in each point prevalence survey in Asia. P50 is shown from all aquatic animal species from all aquatic environments.

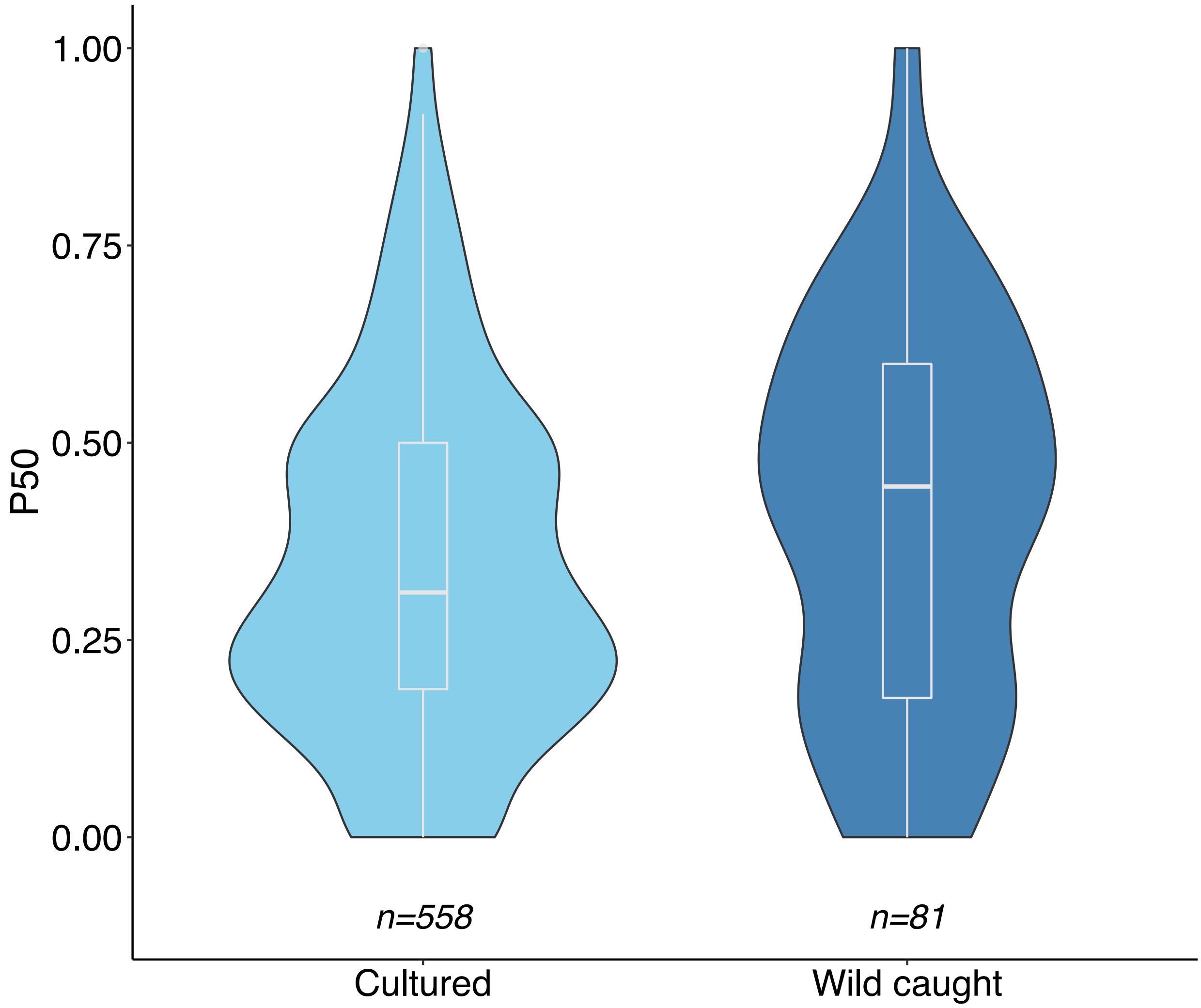

Fig. S7. Distribution of P50 for cultured and wild caught aquatic animals. The horizontal box lines represent the first quartile, the median, and the third quartile. Whiskers denote the range of points within the first quartile − 1.5× the interquartile range and the third quartile + 1.5× the interquartile range. n = 639 individual point prevalence surveys. One-way ANOVA p=.059

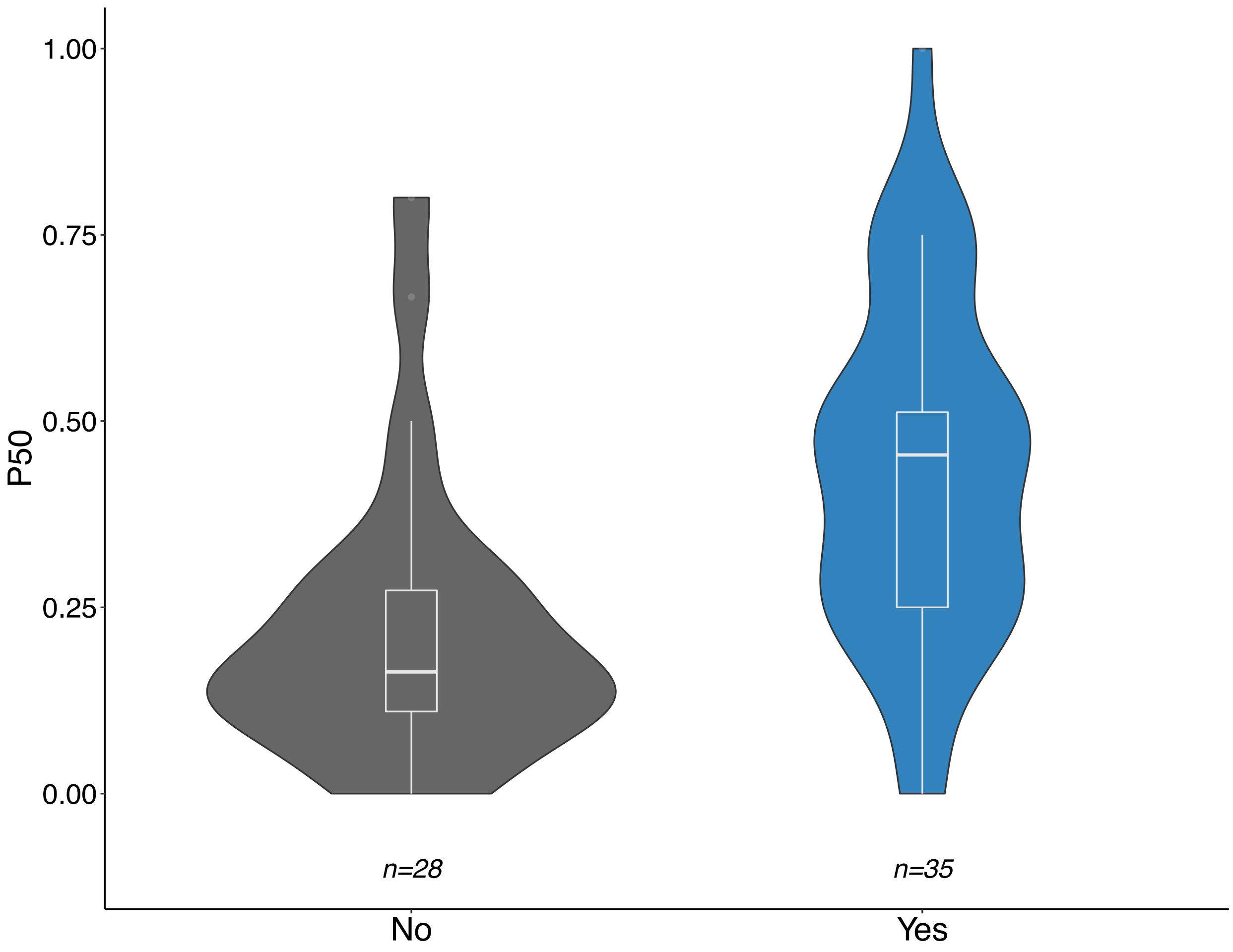

Fig. S8. Distribution of P50 from surveys in cultured aquatic animals in which history of antimicrobial use prior to sampling was recorded. Analysis is limited to surveys that explicitly identified that antimicrobials were ("yes") or were not ("no") applied to the sampled animals prior to sampling (*n* = 63 individual point prevalence surveys). The horizontal box lines represent the first quartile, the median, and the third quartile. Whiskers denote the range of points within the first quartile − 1.5× the interquartile range and the third quartile + 1.5× the interquartile range. One-way ANOVA p=2E-04.

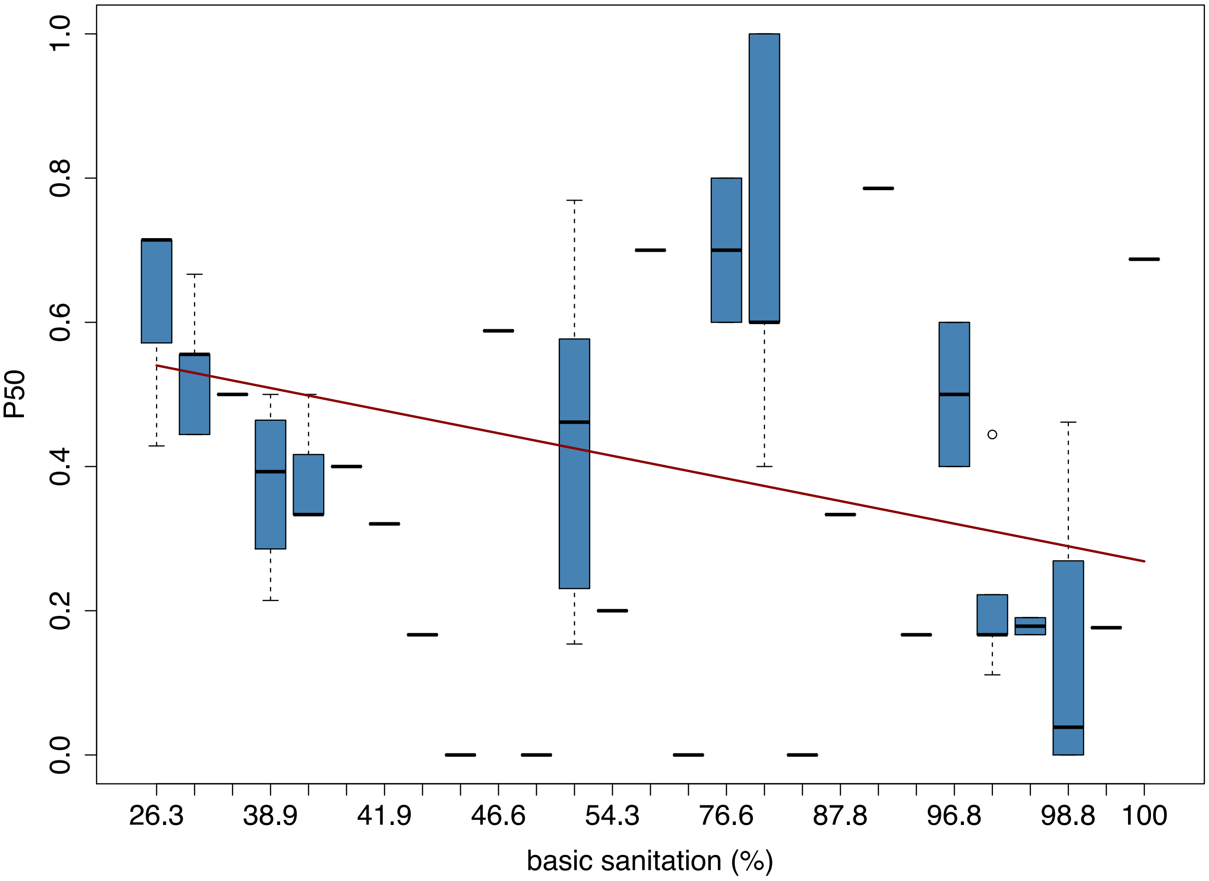

Fig. S9. P50 correlation with World Bank basic sanitation index[18]. The correlation is shown for surveys from wild caught aquatic animals in both freshwater and marine environments (*n* = 80). The horizontal box lines represent the first quartile, the median, and the third quartile. Whiskers denote the range of points within the first quartile − 1.5× the interquartile range and the third quartile + 1.5× the interquartile range. Regression line is fitted using a generalized linear model with a solid line indicating statistical significance (p=0.024).

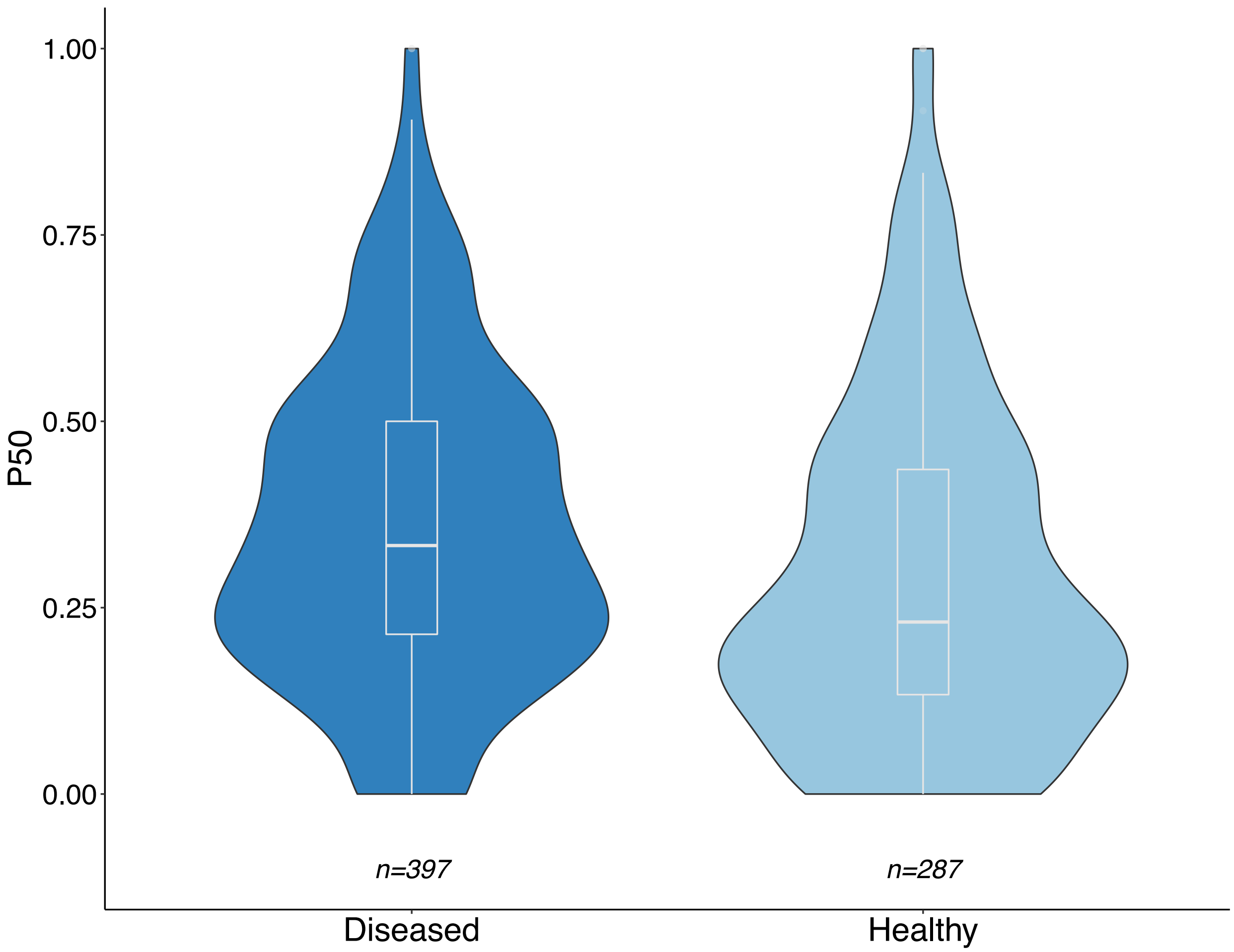

Fig. S10. Distribution of P50 from diseased and healthy animals. The horizontal box lines represent the first quartile, the median, and the third quartile. Whiskers denote the range of points within the first quartile − 1.5× the interquartile range and the third quartile + 1.5× the interquartile range. n = 684 individual point prevalence surveys. One-way ANOVA p=6.05E-05.

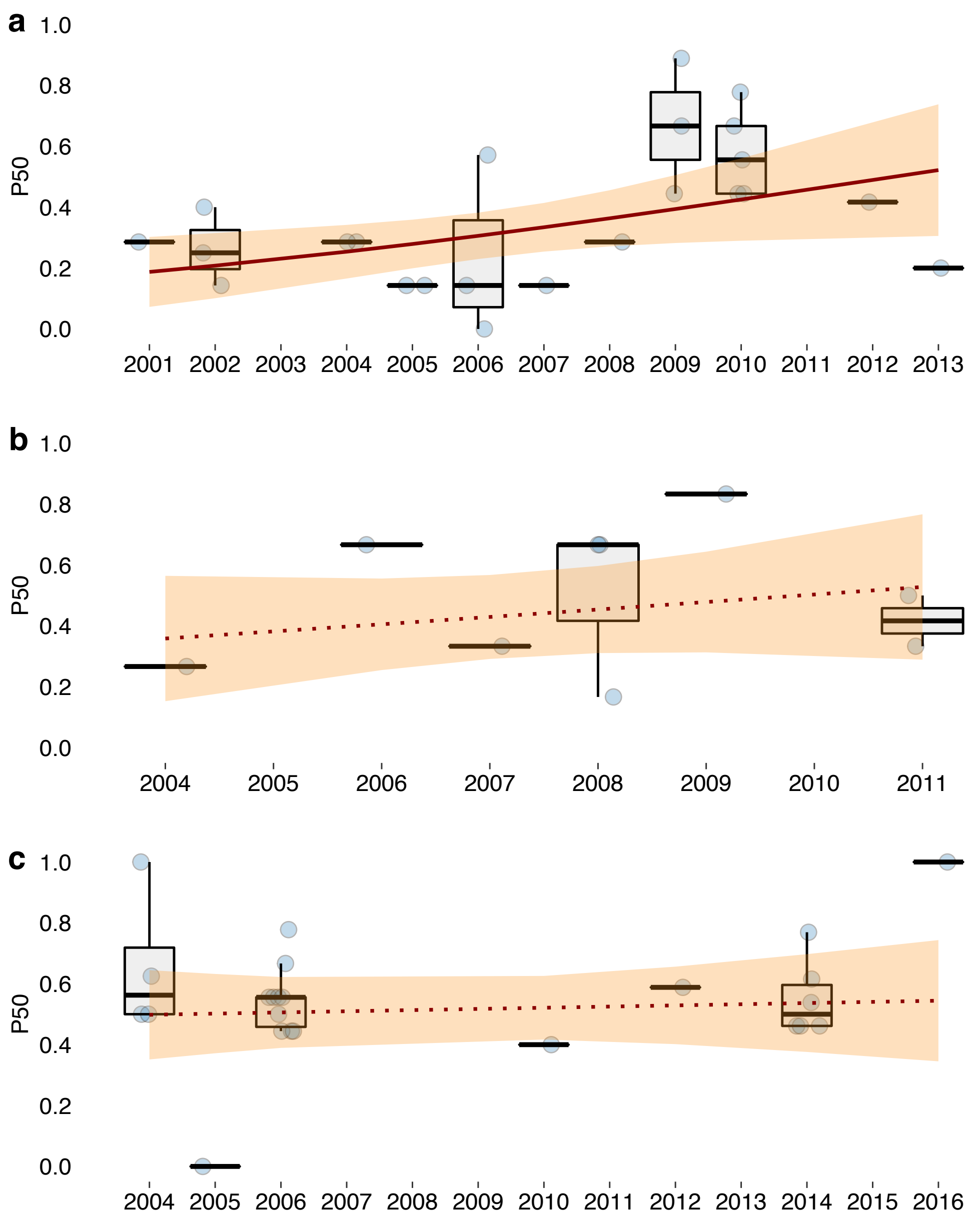

Fig. S11. Annual P50 trends for select pathogen-animal-country pairings. (a) Surveys of *Yersinia* spp. sampled from trout in Turkey (n = 23; p=.049); **(b)** Surveys of *Edwardsiella* spp. sampled from catfish in Vietnam (n = 9; p=.388); and **(c)** Surveys of Enterobacteriaceae sampled from fish in India (n = 24; p=.751). The horizontal box lines represent the first quartile, the median, and the third quartile. Whiskers denote the range of points within the first quartile − 1.5× the interquartile range and the third quartile + 1.5× the interquartile range. Each survey is represented by a dot with horizontal jitter for visibility. Regression lines are fit using generalized linear model regressions, with a solid line in panel (a) indicating statistical significance (p=.049); 95% confidence intervals are shown in shaded areas.

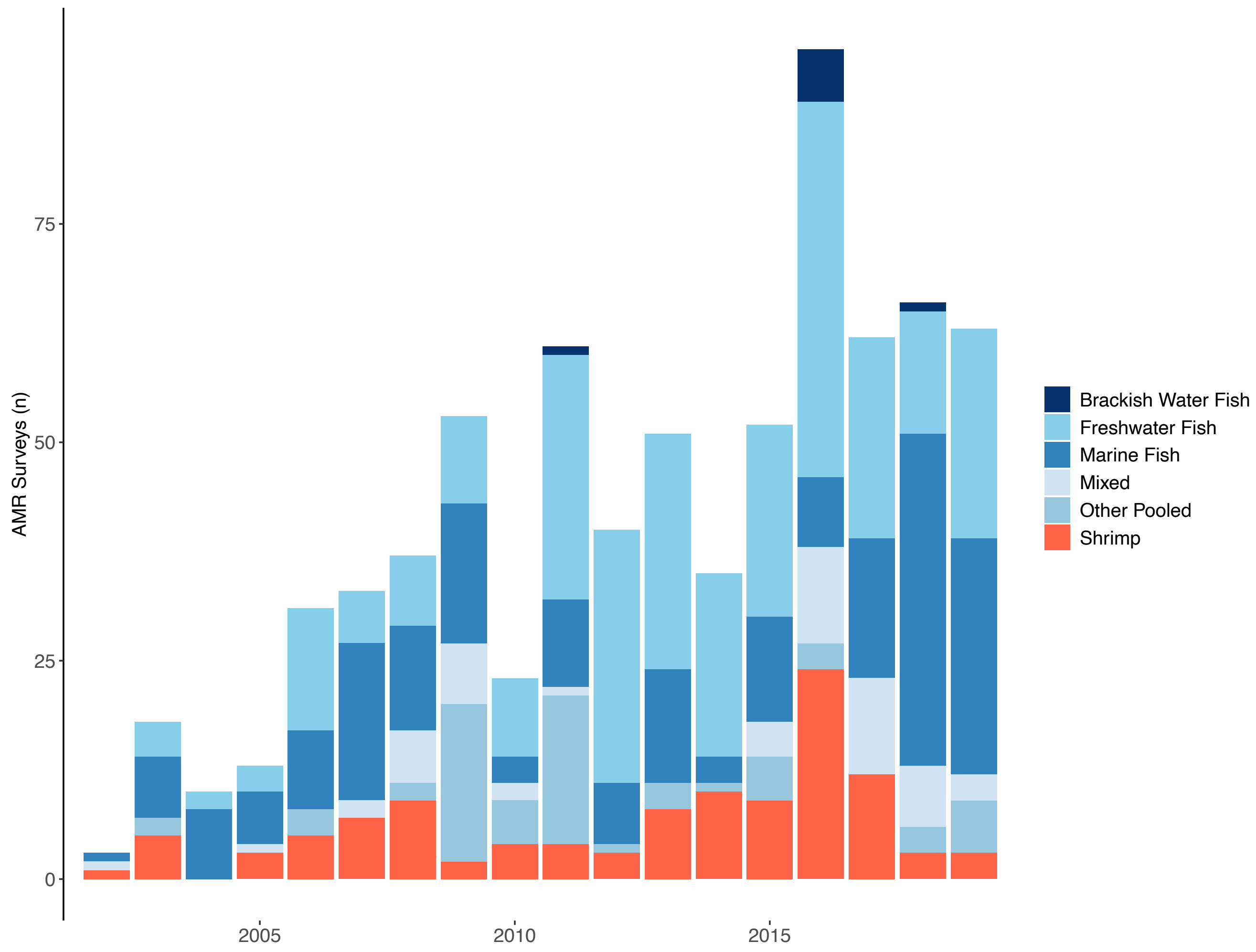

Fig. S12. **Species groups represented in resistance surveys from aquatic animals.** Year of publication is shown.

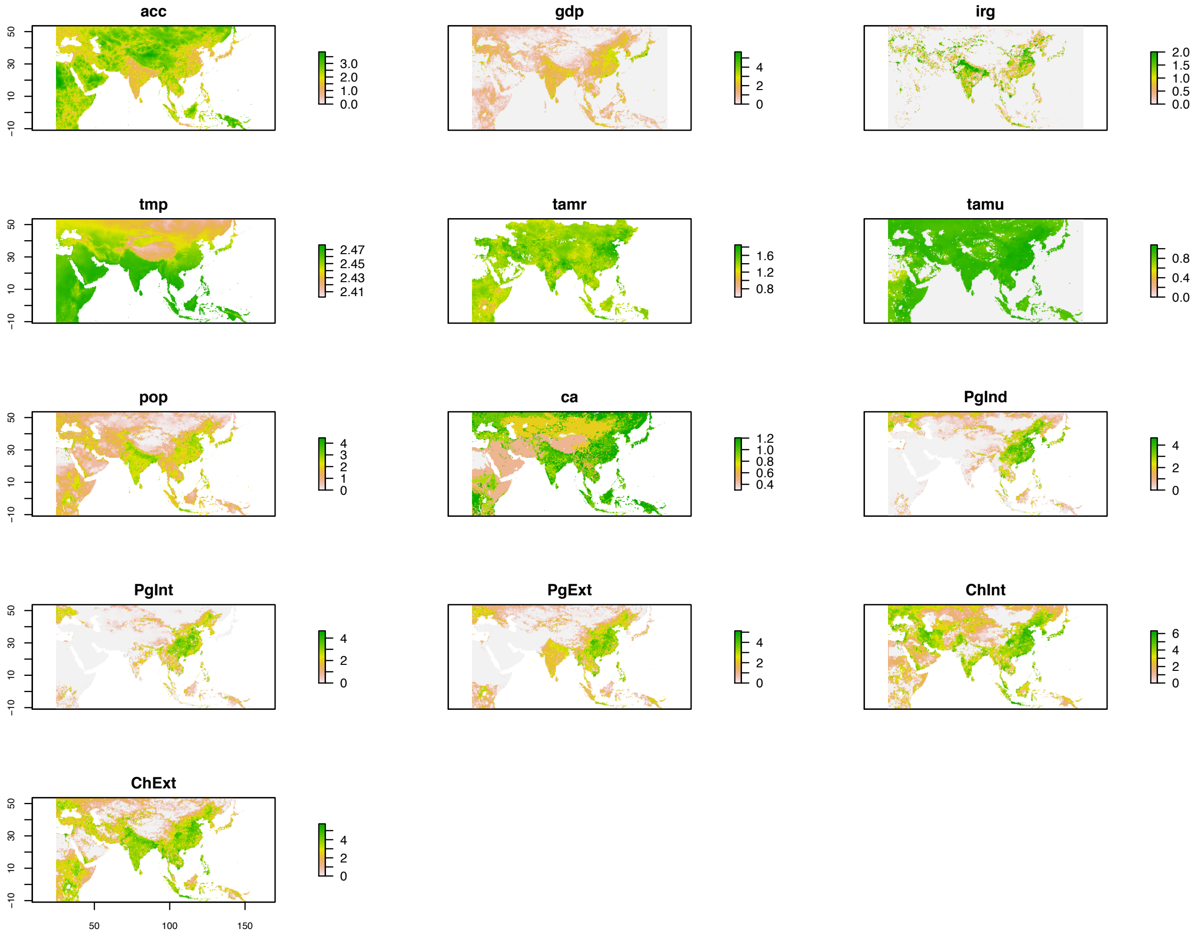

Fig. S13. Freshwater environmental and anthropogenic covariates used to train child models. All covariates are log10 scaled. Accessibility to cities (acc); gross domestic product (gdp); irrigated land percentage (irg); minimum monthly temperature (tmp); terrestrial livestock P50 (tamr); terrestrial livestock antimicrobial use (tamu); human population density (pop); and population densities of cattle (ca), pigs raised in intensively (PgInd), pigs raised semi-intensively (PgInt), pigs raised extensively (PgExt), chickens raised intensively (ChInt), and chickens raised extensively (ChExt). (See Table S2 for details)

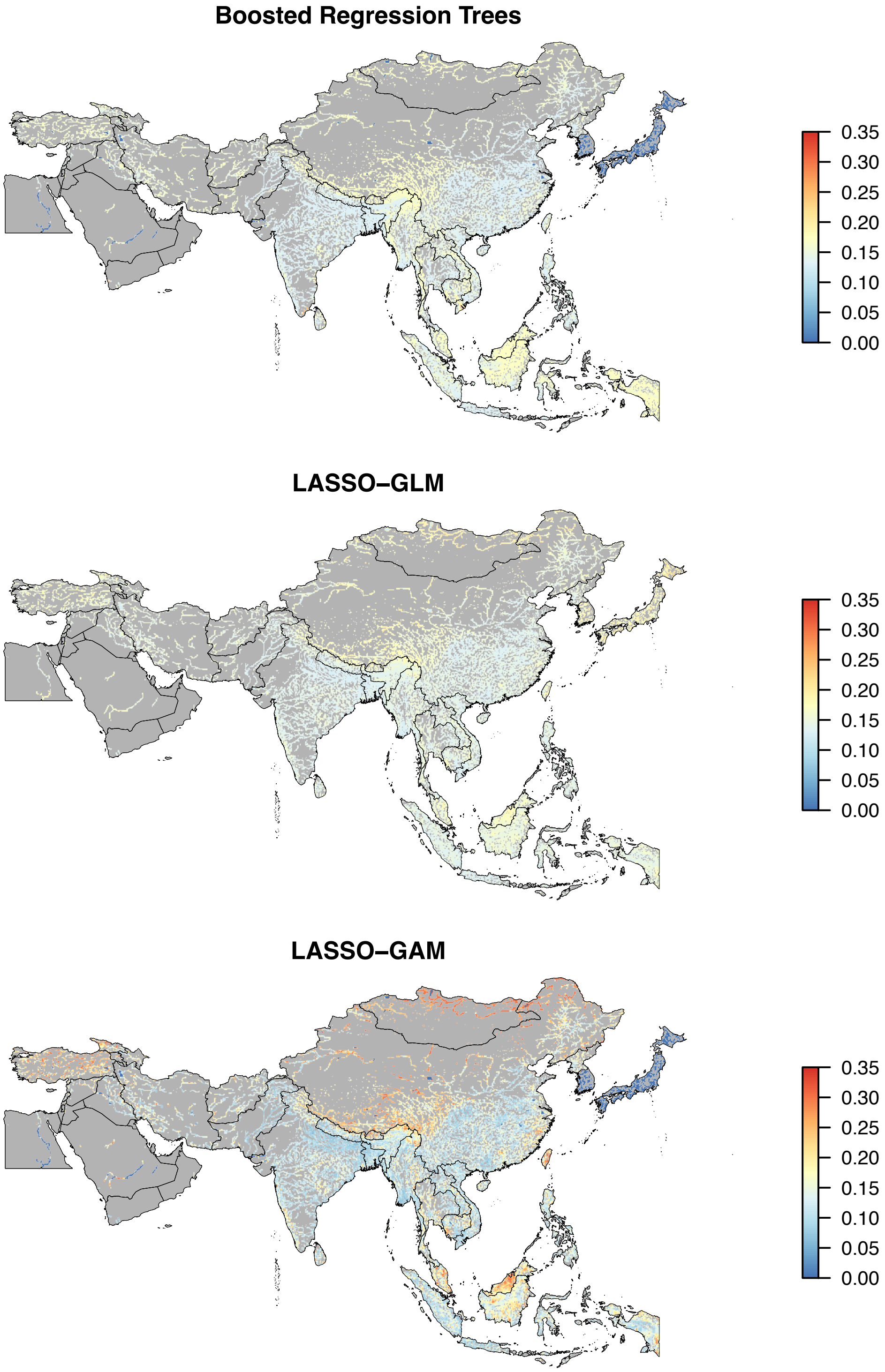

Fig. S14. Freshwater predicted P50 maps for each child model trained to environmental and anthropogenic covariates. Child models are boosted regression trees (BRT); least absolute shrinkage and selection operator applied to logistic regression (LASSO-GLM); and overlapped grouped LASSO penalties for General Additive Models selection (LASSO-GAM).

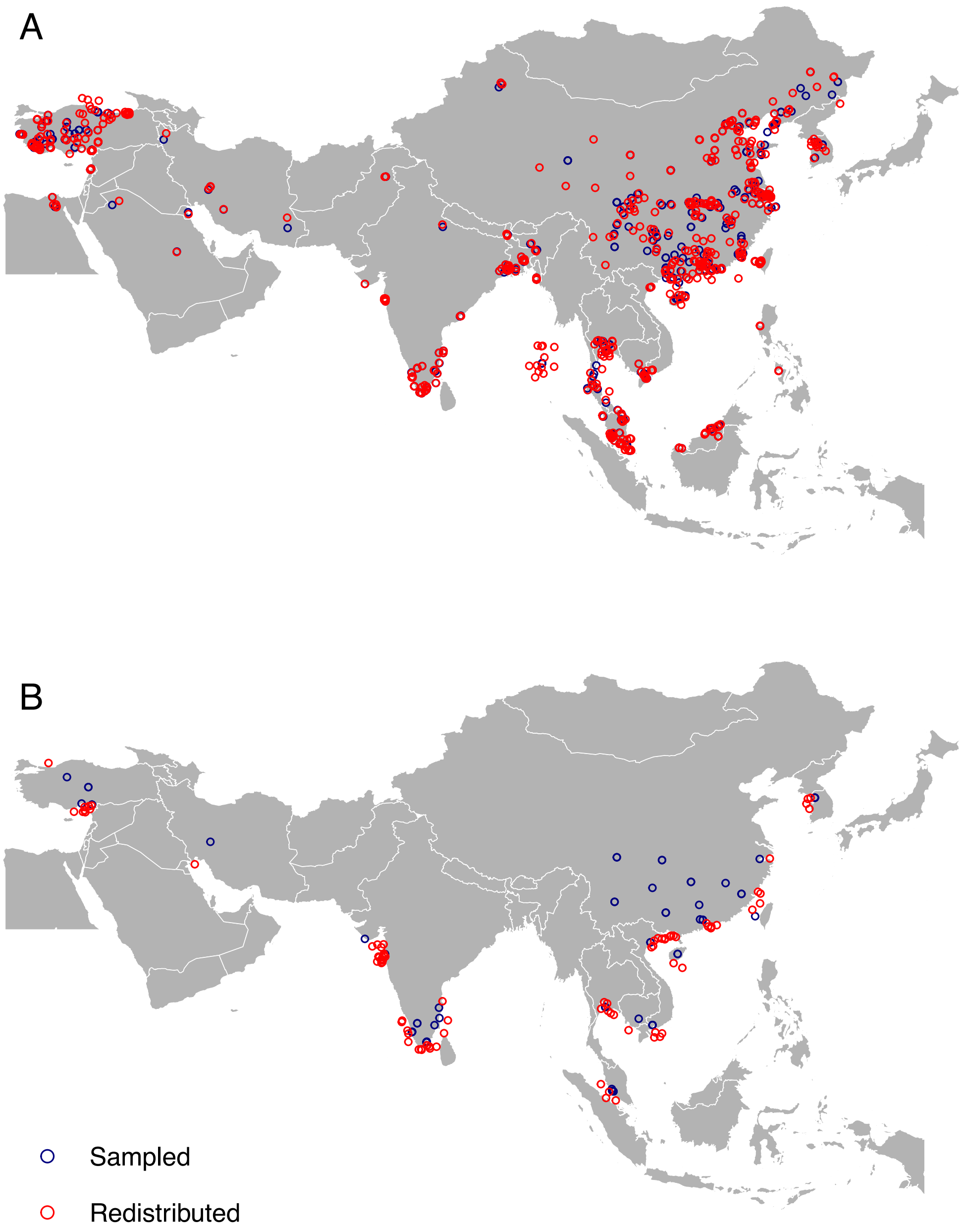

Fig. S15. Redistribution of surveys in (A) freshwater and (B) marine models. Freshwater surveys without precise sampling coordinates were redistributed at random within a geographic uncertainty range of the smallest available administrative unit. Surveys were redistributed on each of 10 bootstrap runs; a single bootstrap run is displayed. In some instances, given sampling locations (blue) are superimposed where multiple surveys were conducted at the same location. In the marine model (B), only post-harvest surveys were redistributed to open water (Supplementary Note 4, Marine Protocol) and are displayed.

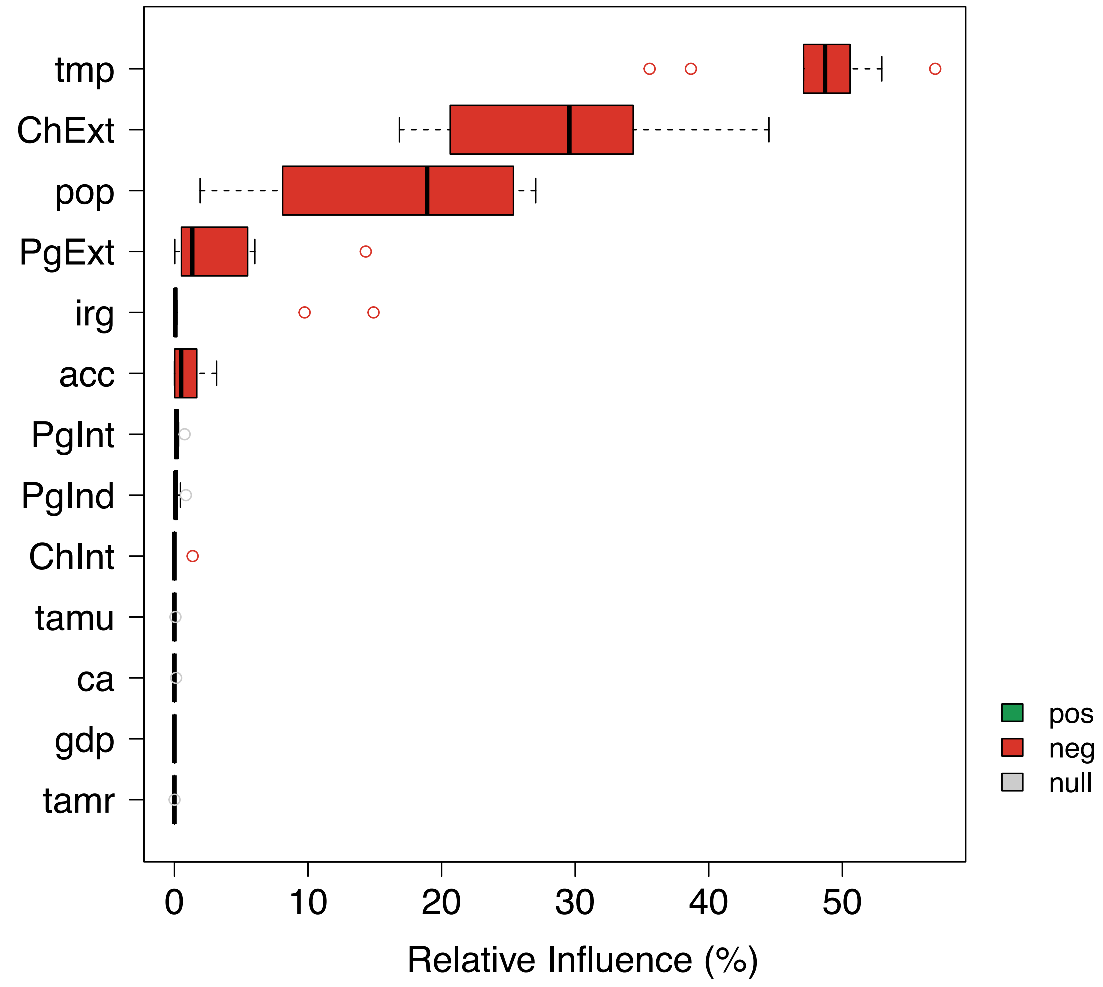

Fig. S16. Relative influence of covariates on resistance in freshwater boosted regression tree (BRT) model. Distributions of relative influence for each covariate in freshwater BRT models across 10 bootstrap runs (*n* = 10; for acronyms, see Supplementary Table S2). Coefficients after regularization from LASSO-GLM models were assigned as positive or negative for each covariate in each run. Positive, negative, and null associations with resistance for each covariate reflect the mean value of bootstrap runs where the coefficients were positive exceeding 30%, where the coefficients were negative, and where the coefficients were positive but in less than 30% of runs, respectively. The vertical box lines represent the first quartile, the median, and the third quartile. Whiskers denote the range of points within the first quartile − 1.5× the interquartile range and the third quartile + 1.5× the interquartile range. No positive associations (coefficients positive exceeding 30%) were identified.

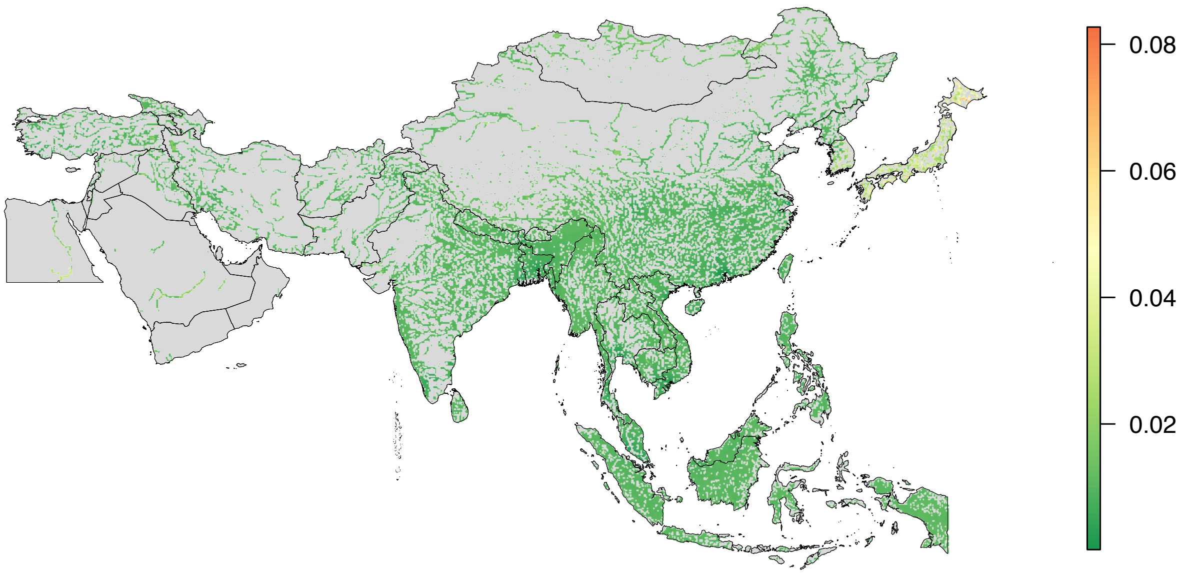

Fig. S17. Standardized kriging variance (spatial interpolation uncertainty) on P50 predictions in freshwater environments. Variance is standardized such that it equals zero at the location of observations.

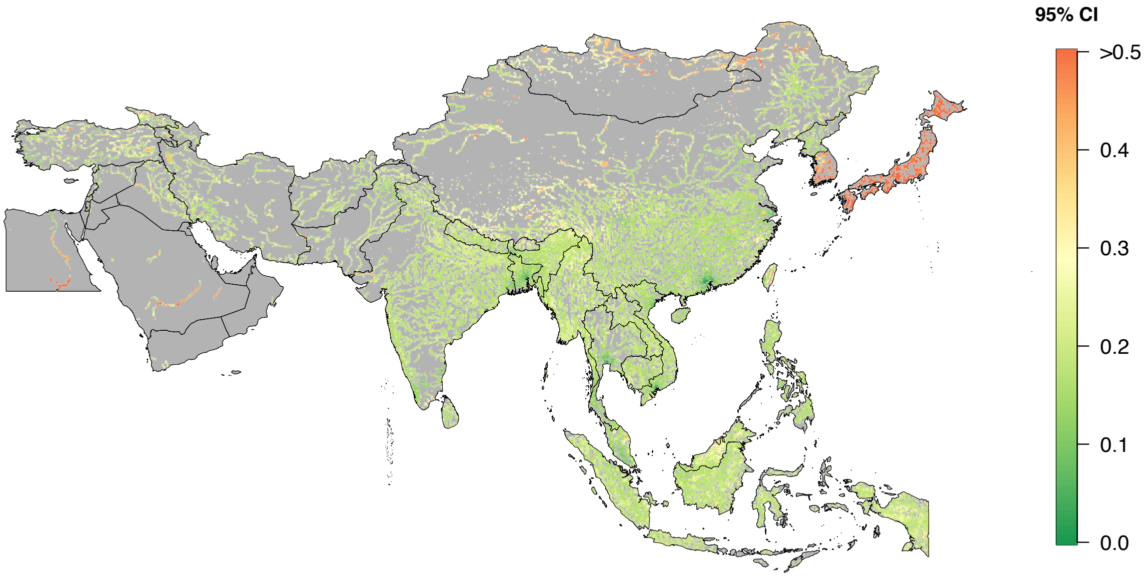

Fig. S18. 95% confidence interval on P50 predictions in freshwater environments.

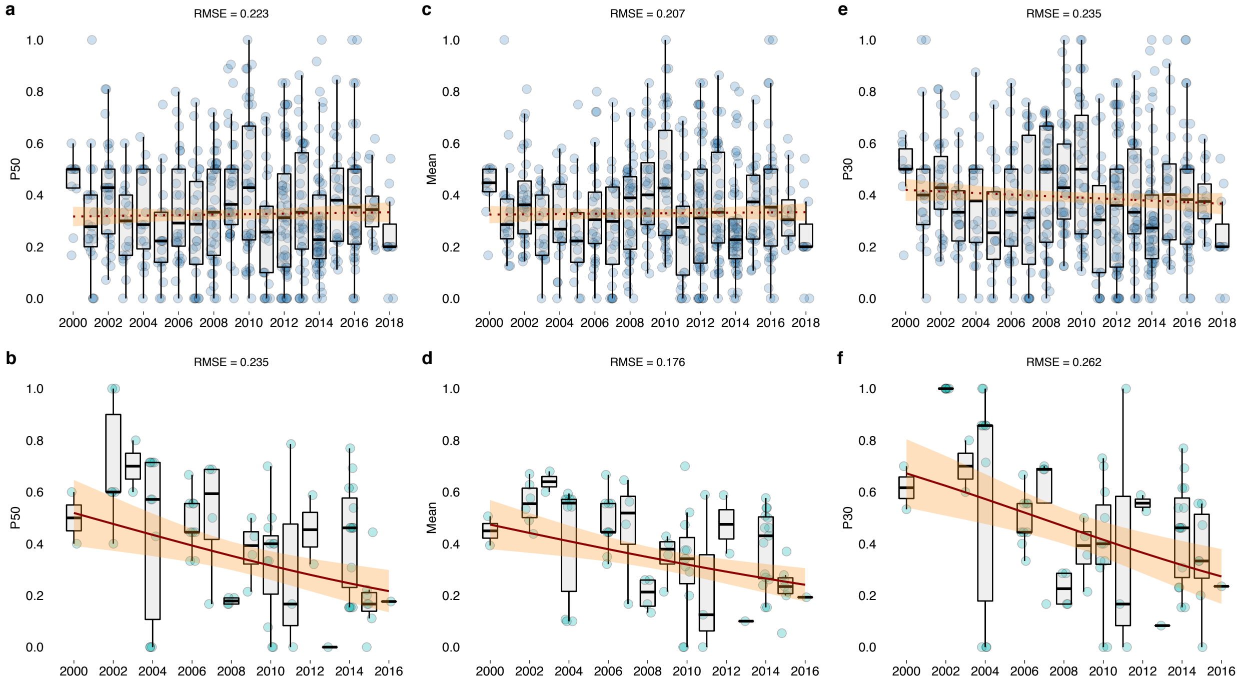

Fig. S19. Annual trends in multi-drug resistance. Surveys from cultured aquatic animals (top row, *n* = 558); surveys from wild caught aquatic animals (bottom row, *n* = 81). **(a,b)** the proportion of drugs with resistance greater than 50% (P50) in each survey; **(c,d)** mean resistance calculated as resistant isolates divided by the number of isolates * number of antibiotics tested in each survey; and **(e,f)** the proportion of drugs with resistance greater than 30% (P30) in each survey. The horizontal box lines represent the first quartile, the median, and the third quartile. Whiskers denote the range of points within the first quartile − 1.5× the interquartile range and the third quartile + 1.5× the interquartile range. Each survey is represented by a dot with horizontal jitter for visibility. Regression lines are fit using generalized linear model regressions, with a solid line indicating statistical significance (**(b)** p=0.003; **(d)** p=0.002; **(f)** p=0.001); 95% confidence intervals are shown in shaded areas. Goodness of fit is assessed using root mean square error (RMSE).

Table S1. Antimicrobial classes and corresponding compounds recommended by The Clinical & Laboratory Standards Institute (CLSI) for susceptibility testing of *Aeromonas* (M45)[19], *Streptococcus* (M100)[20], and *Vibrio* (M45), and by the WHO Advisory Group on Integrated Surveillance of Antimicrobial Resistance (AGISAR) for *E. coli*.[21] *Compounds not represented in the dataset.

**Antimicrobial Class**	*Aeromonas* spp.	*E. coli*	*Streptococcus* spp. (β-Hemolytic /Viridans)	*Vibrio* spp.
Aminoglycosides	Amikacin<br>Gentamicin	Gentamicin		Amikacin<br>Gentamicin
Amphenicols	Chloramphenicol	Chloramphenicol	Chloramphenicol	Chloramphenicol
Carbapenems	Ertapenem<br>Imipenem<br>Meropenem	Imipenem<br>Meropenem	Ertapenem<br>Meropenem	Imipenem<br>Meropenem
Cephalosporins	Cefepime<br>Cefotaxime<br>Cefoxitin<br>Ceftazidime<br>Ceftriaxone<br>Cefuroxime	Cefepime<br>Cefotaxime<br>Cefoxitin<br>Ceftazidime<br>Ceftriaxone	Cefepime<br>Cefotaxime<br>Ceftaroline*<br>Ceftriaxone	Cefazolin<br>Cefepime<br>Cefotaxime<br>Cefoxitin<br>Ceftazidime<br>Cefuroxime
Glycopeptides			Vancomycin	
Glycylcyclines		Tigecycline*		
Lincosamides			Clindamycin	
Lipopeptides			Daptomycin*	
Macrolides		Azithromycin	Azithromycin<br>Clarithromycin<br>Erythromycin	Azithromycin
Monobactams	Aztreonam			
Nitrofurans		Nitrofurantoin		
Oxazolidinones			Linezolid	
Penicillins	Piperacillin-Tazobactam	Amoxicillin<br>Ampicillin<br>Temocillin*	Ampicillin<br>Penicillin	Amoxicillin-Clavulanic Acid<br>Ampicillin<br>Ampicillin-Sulbactam<br>Piperacillin<br>Piperacillin-Tazobactam
Polymyxins		Colistin		
Quinolones	Ciprofloxacin<br>Levofloxacin	Ciprofloxacin<br>Nalidixic acid<br>Pefloxacin	Levofloxacin<br>Ofloxacin	Ciprofloxacin<br>Levofloxacin<br>Ofloxacin
Sulfonamides and dihydrofolate reductase inhibitors	Sulfamethoxazole-Trimethoprim	Sulfamethoxazole<br>Sulfamethoxazole-Trimethoprim<br>Sulfamonomethoxine<br>Sulfisoxazole<br>Sulfonamides<br>Trimethoprim		Sulfadiazine<br>Sulfamethoxazole<br>Sulfamethoxazole-Trimethoprim<br>Sulfamonomethoxine<br>Sulfisoxazole<br>Sulfonamides<br>Trimethoprim

				Trimethoprim-Sulfadiazine
Tetracyclines	Tetracycline	Tetracycline	Tetracycline	Doxycycline<br>Tetracycline

Table S2. Freshwater environmental and anthropogenic covariates used to train child models.

Name	Acronym	Year	Resolution	Source
Accessibility to Cities	acc	2015	30 arc second	Weiss D et al. Accessibility to Cities https://malariaatlas.org/research-project/accessibility_to_cities/
Gross Domestic Product	gdp	2010	30 arc second	World Bank https://datacatalog.worldbank.org/dataset/gross-domestic-product-2010
Irrigated Land Percentage	irg	2005	0.083333 decimal degrees	Siebert S et al. Global Map of Irrigation Areas http://www.fao.org/aquastat/en/geospatial-information/global-maps-irrigated-areas/latest-version
Minimum Monthly Temperature	tmp	2020	2.5 minutes	WorldClim https://www.worldclim.org/data/worldclim21.html
Terrestrial Livestock P50	amr	2018	0.083333 decimal degrees	Van Boeckel T et al. https://resistancebank.org
Terrestrial Livestock AMU	amu	2017	0.083333 decimal degrees	Van Boeckel T et al.
Human Population Density	pop	2020	2.5 minutes	Center for International Earth Science Information Network - CIESIN - Columbia University. 2018. Gridded Population of the World, Version 4 (GPWv4): Population Density, Revision 11. https://sedac.ciesin.columbia.edu/data/set/gpw-v4-population-density-rev11
Terrestrial Livestock Production	Ca<br>PgInd<br>PgInt<br>PgExt<br>ChInt<br>ChExt	2010	0.083333 decimal degrees	Robinson TP et al. Global distribution of ruminant livestock production systems V5 (5 minutes of arc)<br><br>Gilbert M et al. Global distribution of chickens and pigs raised in extensive, semi-intensive and intensive systems in 2010 (5 minutes of arc)<br><br>http://www.fao.org/livestock-systems/production-systems/en/

Table S3. Root mean square error (RMSE) weightings in the marine ensemble model. Weights are calculated as the inverse of the RMSE of each constituent model divided by the sum of RMSE for all models [Weights = $1/(RMSE_i/\sum_{i=1}^{3} RMSE_i)$], and expressed as their relative proportion.

	RMSE	Model weights
Inverse distance weighted	0.2359776	31.7
Natural neighbor	0.2255336	33.2
Ordinary kriging	0.2131811	35.1

Table S4. Preferred Reporting Items for Systematic Reviews and Meta-Analyses (PRISMA) 2020 main checklist.

Topic	No.	Item	Location where item is reported
**TITLE**			
**Title**	1	Identify the report as a systematic review.	Introduction, Line 105
**ABSTRACT**			
**Abstract**	2	See the PRISMA 2020 for Abstracts checklist	
**INTRODUCTION**			
**Rationale**	3	Describe the rationale for the review in the context of existing knowledge.	Line 94-108
**Objectives**	4	Provide an explicit statement of the objective(s) or question(s) the review addresses.	Line 105-106
**METHODS**			
**Eligibility criteria**	5	Specify the inclusion and exclusion criteria for the review and how studies were grouped for the syntheses.	Line 392-396; SI Line 33-45
**Information sources**	6	Specify all databases, registers, websites, organisations, reference lists and other sources searched or consulted to identify studies. Specify the date when each source was last searched or consulted.	Line 389-391; SI Line 50-55
**Search strategy**	7	Present the full search strategies for all databases, registers and websites, including any filters and limits used.	SI Line 65-161
**Selection process**	8	Specify the methods used to decide whether a study met the inclusion criteria of the review, including how many reviewers screened each record and each report retrieved, whether they worked independently, and if applicable, details of automation tools used in the process.	SI Line 45-48
**Data collection process**	9	Specify the methods used to collect data from reports, including how many reviewers collected data from each report, whether they worked independently, any processes for obtaining or confirming data from study investigators, and if applicable, details of automation tools used in the process.	Line 400-411; SI Line 167-180; legend

Topic	No.	Item	Location where item is reported
**Data items**	10a	List and define all outcomes for which data were sought. Specify whether all results that were compatible with each outcome domain in each study were sought (e.g. for all measures, time points, analyses), and if not, the methods used to decide which results to collect.	Line 400-411; SI Line 167-180; legend
	10b	List and define all other variables for which data were sought (e.g. participant and intervention characteristics, funding sources). Describe any assumptions made about any missing or unclear information.	Line 407-411; SI Line 238-245; legend
**Study risk of bias assessment**	11	Specify the methods used to assess risk of bias in the included studies, including details of the tool(s) used, how many reviewers assessed each study and whether they worked independently, and if applicable, details of automation tools used in the process.	SI Line 45-48
**Effect measures**	12	Specify for each outcome the effect measure(s) (e.g. risk ratio, mean difference) used in the synthesis or presentation of results.	Line 428-430; 452-453
**Synthesis methods**	13a	Describe the processes used to decide which studies were eligible for each synthesis (e.g. tabulating the study intervention characteristics and comparing against the planned groups for each synthesis (item 5)).	Line 391-394
	13b	Describe any methods required to prepare the data for presentation or synthesis, such as handling of missing summary statistics, or data conversions.	Line 413-425
	13c	Describe any methods used to tabulate or visually display results of individual studies and syntheses.	Line 436-443; 452-453
	13d	Describe any methods used to synthesize results and provide a rationale for the choice(s). If meta-analysis was performed, describe the model(s), method(s) to identify the presence and extent of statistical heterogeneity, and software package(s) used.	Line 436-440; 445-453; SI Line 259-321; 458
	13e	Describe any methods used to explore possible causes of heterogeneity among study results (e.g. subgroup analysis, meta-regression).	Line 436-441

Topic	No.	Item	Location where item is reported
	13f	Describe any sensitivity analyses conducted to assess robustness of the synthesized results.	Line 430-435; 348-351; SI Line 272-283
**Reporting bias assessment**	14	Describe any methods used to assess risk of bias due to missing results in a synthesis (arising from reporting biases).	Line 407-411; SI Line 45-48
**Certainty assessment**	15	Describe any methods used to assess certainty (or confidence) in the body of evidence for an outcome.	Line 441-443;461; 522-524
**RESULTS**			
**Study selection**	16a	Describe the results of the search and selection process, from the number of records identified in the search to the number of studies included in the review, ideally using a flow diagram.	Line 112-113; SI Line 163-165; Fig. S1
	16b	Cite studies that might appear to meet the inclusion criteria, but which were excluded, and explain why they were excluded.	Supplementary data 2; Fig. S1
**Study characteristics**	17	Cite each included study and present its characteristics.	Supplementary data 2
**Risk of bias in studies**	18	Present assessments of risk of bias for each included study.	At screening/eligibility; Fig. S1
**Results of individual studies**	19	For all outcomes, present, for each study: (a) summary statistics for each group (where appropriate) and (b) an effect estimate and its precision (e.g. confidence/credible interval), ideally using structured tables or plots.	Supplementary data 1; Fig. 1; Fig. 2
**Results of syntheses**	20a	For each synthesis, briefly summarise the characteristics and risk of bias among contributing studies.	Line 112-119
	20b	Present results of all statistical syntheses conducted. If meta-analysis was done, present for each the summary estimate and its precision (e.g. confidence/credible interval) and measures of statistical heterogeneity. If comparing groups, describe the direction of the effect.	Line 121-157
	20c	Present results of all investigations of possible causes of heterogeneity among study results.	Line 439-441
	20d	Present results of all sensitivity analyses conducted to assess the robustness of the synthesized results.	SI Line 272-283; Fig. S19

Topic	No.	Item	Location where item is reported
**Reporting biases**	21	Present assessments of risk of bias due to missing results (arising from reporting biases) for each synthesis assessed.	Line 112-119; 359-366
**Certainty of evidence**	22	Present assessments of certainty (or confidence) in the body of evidence for each outcome assessed.	Line 122-157; 168-169; Fig. 1; Fig. 2; Fig. S18
**DISCUSSION**			
**Discussion**	23a	Provide a general interpretation of the results in the context of other evidence.	Line 191-203
	23b	Discuss any limitations of the evidence included in the review.	Line 330-362
	23c	Discuss any limitations of the review processes used.	Line 363-366
	23d	Discuss implications of the results for practice, policy, and future research.	Line 372-384
**OTHER INFORMATION**			
**Registration and protocol**	24a	Provide registration information for the review, including register name and registration number, or state that the review was not registered.	SI Line 55
	24b	Indicate where the review protocol can be accessed, or state that a protocol was not prepared.	SI Line 179-180
	24c	Describe and explain any amendments to information provided at registration or in the protocol.	n/a
**Support**	25	Describe sources of financial or non-financial support for the review, and the role of the funders or sponsors in the review.	Line 729-731
**Competing interests**	26	Declare any competing interests of review authors.	Line 738-739
**Availability of data, code and other materials**	27	Report which of the following are publicly available and where they can be found: template data collection forms; data extracted from included studies; data used for all analyses; analytic code; any other materials used in the review.	Supplementary data 1; Line 564-570

Supplementary References

1. International Office of Epizootics. *Aquatic animal health code.* (2019).
2. Page, M. J. *et al.* The PRISMA 2020 statement: An updated guideline for reporting systematic reviews. *PLOS Med.* **18**, e1003583 (2021).
3. Van Boeckel, T. P. *et al.* Global trends in antimicrobial resistance in animals in low- and middle-income countries. *Science* **365**, eaaw1944 (2019).
4. Schar, Daniel *et al.* Aquatic animal antimicrobial resistance point prevalence survey database legend. (2021) doi:10.5281/ZENODO.4609884.
5. CLSI. *Performance Standards for Antimicrobial Susceptibility Testing of Bacteria Isolated From Aquatic Animals.* vol. CLSI supplement VET04. (Clinical and Laboratory Standards Institute, 2020).
6. U.S. FDA. The National Antimicrobial Resistance Monitoring System: Strategic Plan 2021 - 2025. (2020).
7. Golding, N. *et al.* Mapping under-5 and neonatal mortality in Africa, 2000–15: a baseline analysis for the Sustainable Development Goals. *The Lancet* **390**, 2171–2182 (2017).
8. Bhatt, S. *et al.* Improved prediction accuracy for disease risk mapping using Gaussian process stacked generalization. *J. R. Soc. Interface* **14**, 20170520 (2017).
9. Barbet-Massin, M., Jiguet, F., Albert, C. H. & Thuiller, W. Selecting pseudo-absences for species distribution models: how, where and how many?: *How to use pseudo-absences in niche modelling? Methods Ecol. Evol.* **3**, 327–338 (2012).
10. Elith, J., Leathwick, J. R. & Hastie, T. A working guide to boosted regression trees. *J. Anim. Ecol.* **77**, 802–813 (2008).
11. Tibshirani, R. Regression Shrinkage and Selection Via the Lasso. *J. R. Stat. Soc. Ser. B Methodol.* **58**, 267–288 (1996).
12. Chouldechova, A. & Hastie, T. Generalized Additive Model Selection. *ArXiv150603850 Stat* (2015).

13. Hijmans, R. J. Cross-validation of species distribution models: removing spatial sorting bias and calibration with a null model. *Ecology* **93**, 679–688 (2012).

14. Lehner, B. & Döll, P. Development and validation of a global database of lakes, reservoirs and wetlands. *J. Hydrol.* **296**, 1–22 (2004).

15. Messager, M. L., Lehner, B., Grill, G., Nedeva, I. & Schmitt, O. Estimating the volume and age of water stored in global lakes using a geo-statistical approach. *Nat. Commun.* **7**, 13603 (2016).

16. Lehner, B. & Grill, G. Global river hydrography and network routing: baseline data and new approaches to study the world's large river systems: GLOBAL RIVER HYDROGRAPHY AND NETWORK ROUTING. *Hydrol. Process.* **27**, 2171–2186 (2013).

17. Toni, T., Welch, D., Strelkowa, N., Ipsen, A. & Stumpf, M. P. H. Approximate Bayesian computation scheme for parameter inference and model selection in dynamical systems. *J. R. Soc. Interface* **6**, 187–202 (2009).

18. World Bank. *People using at least basic sanitation services (% of population)*. data.worldbank.org.

19. *Methods for antimicrobial dilution and disk susceptibility testing of infrequently isolated or fastidious bacteria*. (Clinical and Laboratory Standards Institute, 2016).

20. WEINSTEIN, M. P. *M100-performance standards for antimicrobial susceptibility testing, 28th edition.* (CLINICAL AND LABORATORY, 2018).

21. WHO Advisory Group on Integrated Surveillance of Antimicrobial Resistance & World Health Organization. *Critically important antimicrobials for human medicine: ranking of antimicrobial agents for risk management of antimicrobial resistance due to non-human use*. (2017).
