# Supplementary material for: Twenty-year trends in antimicrobial resistance from aquaculture and fisheries in Asia
Source: Nat Commun. 2021 Sep 10;12:5384. doi: 10.1038/s41467-021-25655-8 (PMC8433129; doi:10.1038/s41467-021-25655-8)
Supplement: Supplementary file 3 — Description of Additional Supplementary Files [file 41467_2021_25655_MOESM3_ESM.pdf]

Description of Additional Supplementary Files

File Name: Supplementary Data 1
Description: Point prevalence survey data from the systematic review are provided in Supplementary Data 1.

File Name: Supplementary Data 2
Description: Records included and excluded from the systematic review are provided in Supplementary Data 2.

File Name: Supplementary Software 1
Description: This file contains R code used to analyze the data and generate the models.
