# Supplementary material for: Twenty-year trends in antimicrobial resistance from aquaculture and fisheries in Asia
Source: Nat Commun. 2021 Sep 10;12:5384. doi: 10.1038/s41467-021-25655-8 (PMC8433129; doi:10.1038/s41467-021-25655-8)
Supplement: Supplementary file 6 — Supplementary Software [file 41467_2021_25655_MOESM6_ESM.zip › R scripts__NCOMMS-21-08900/readme.rtf]

All analyses conducted in R version 3.6.3R is freely available, open source at https://cran.r-project.org/mirrors.htmlPackages required to run scripts are identified in each script and are freely available, open source. No non-standard hardware is required to run scripts.Each script includes detailed instructions for use with the accompanying dataset and expected outputs. Run times for geospatial models and raster processing on standard computers can be lengthy (1-2 hours).
