# Supplementary material for: Twenty-year trends in antimicrobial resistance from aquaculture and fisheries in Asia
Source: Nat Commun. 2021 Sep 10;12:5384. doi: 10.1038/s41467-021-25655-8 (PMC8433129; doi:10.1038/s41467-021-25655-8)
Supplement: Supplementary file 7 — Reporting Summary [file 41467_2021_25655_MOESM7_ESM.pdf]

nature portfolio

Corresponding author(s): Daniel Schar

Last updated by author(s): Aug 12, 2021

Reporting Summary

Nature Portfolio wishes to improve the reproducibility of the work that we publish. This form provides structure for consistency and transparency in reporting. For further information on Nature Portfolio policies, see our Editorial Policies and the Editorial Policy Checklist.

Statistics

For all statistical analyses, confirm that the following items are present in the figure legend, table legend, main text, or Methods section.

n/a	Confirmed	
☐	☒	The exact sample size (n) for each experimental group/condition, given as a discrete number and unit of measurement
☒	☐	A statement on whether measurements were taken from distinct samples or whether the same sample was measured repeatedly
☐	☒	The statistical test(s) used AND whether they are one- or two-sided<br>*Only common tests should be described solely by name; describe more complex techniques in the Methods section.*
☐	☒	A description of all covariates tested
☐	☒	A description of any assumptions or corrections, such as tests of normality and adjustment for multiple comparisons
☐	☒	A full description of the statistical parameters including central tendency (e.g. means) or other basic estimates (e.g. regression coefficient) AND variation (e.g. standard deviation) or associated estimates of uncertainty (e.g. confidence intervals)
☐	☒	For null hypothesis testing, the test statistic (e.g. F, t, r) with confidence intervals, effect sizes, degrees of freedom and P value noted<br>*Give P values as exact values whenever suitable.*
☐	☒	For Bayesian analysis, information on the choice of priors and Markov chain Monte Carlo settings
☒	☐	For hierarchical and complex designs, identification of the appropriate level for tests and full reporting of outcomes
☐	☒	Estimates of effect sizes (e.g. Cohen's d, Pearson's r), indicating how they were calculated

Our web collection on statistics for biologists contains articles on many of the points above.

Software and code

Policy information about availability of computer code

Data collection	Google Sheets and Microsoft Excel (version 16.51)
Data analysis	Custom code in R version 3.6.3 (10.5281/zenodo.4615703)

For manuscripts utilizing custom algorithms or software that are central to the research but not yet described in published literature, software must be made available to editors and reviewers. We strongly encourage code deposition in a community repository (e.g. GitHub). See the Nature Portfolio guidelines for submitting code & software for further information.

Data

Policy information about availability of data

All manuscripts must include a data availability statement. This statement should provide the following information, where applicable:

- Accession codes, unique identifiers, or web links for publicly available datasets
- A description of any restrictions on data availability
- For clinical datasets or third party data, please ensure that the statement adheres to our policy

Datasets generated and analyzed from this study are available on the Zenodo public repository: https://doi.org/10.5281/zenodo.4615703.
The literature search was conducted across four databases (PubMed [https://pubmed.ncbi.nlm.nih.gov], Web of Science [https://login.webofknowledge.com], Scopus [https://www.scopus.com], China National Knowledge Infrastructure [https://www.cnki.net]) and grey literature repositories (AGRIS [https://agris.fao.org/agris-search/index.do], CGIAR FISH [https://fish.cgiar.org/publications], IFPRI [https://www.ifpri.org/publications], WorldFish [https://www.worldfishcenter.org/publications]). The Russian Science Citation Index and Korean Journal Database (KCI) were included in the Web of Science search.

Field-specific reporting

Please select the one below that is the best fit for your research. If you are not sure, read the appropriate sections before making your selection.

- [x] Life sciences
- [ ] Behavioural & social sciences
- [ ] Ecological, evolutionary & environmental sciences

For a reference copy of the document with all sections, see nature.com/documents/nr-reporting-summary-flat.pdf

Life sciences study design

All studies must disclose on these points even when the disclosure is negative.

Sample size	This study is a systematic review and meta-analysis. The meta-analysis is conducted using the dataset of point prevalence surveys generated for this study. The dataset represents 343 records yielding 749 point prevalence surveys and from which 12,698 resistance rates representing 11,289 isolates were extracted.
Data exclusions	Pre-established exclusion criteria were developed for the systematic review of point prevalence surveys (PPS). Specifically, the following were excluded (supplementary information Fig. S1): reviews; meta-analyses; strain surveys describing individual strain characteristics not associated with a PPS; data from an experimental protocol not associated with a PPS; PPS with samples originating from bivalve molluscs; PPS with samples originating from ornamental fish; and PPS where no source or methodology for derivation of data was provided.
Replication	Replication of findings were independently verified (temporal trends and AMR in food borne pathogens-once; geospatial modeling and optimization for future surveillance - twice) by co-authors (DS, TPVB, and CZ) using custom code and the dataset generated for this study.
Randomization	This study is a systematic review and meta-analysis. Analyses were conducted on the data extracted from the dataset of point prevalence surveys (PPS) identified in the systematic review. All surveys with data pertaining to the analyses were included. In the geospatial modeling, we randomized binarization of P50 values and sampling of pseudo-absence points. We also randomly distributed surveys of marine wild caught animals sampled at land based post-harvest sites to open ocean (Supplementary Note 4: Geospatial modeling).
Blinding	This study is a systematic review and meta-analysis. The literature search and systematic review were guided by the Preferred Reporting Items for Systematic reviews and Meta-Analyses (PRISMA) statement and research synthesis norms. Blinding was not a pre-identified component of the literature search and systematic review (protocols detailed in the database legend [doi: 10.5281/zenodo.4609884]), and no blinding of authors to literature search results was undertaken.

Reporting for specific materials, systems and methods

We require information from authors about some types of materials, experimental systems and methods used in many studies. Here, indicate whether each material, system or method listed is relevant to your study. If you are not sure if a list item applies to your research, read the appropriate section before selecting a response.

Materials & experimental systems

n/a	Involved in the study	
x		Antibodies
x		Eukaryotic cell lines
x		Palaeontology and archaeology
x		Animals and other organisms
x		Human research participants
x		Clinical data
x		Dual use research of concern

Methods

n/a	Involved in the study	
x		ChIP-seq
x		Flow cytometry
x		MRI-based neuroimaging
